# An exomoon survey of 70 cool giant exoplanets and the new candidate Kepler-1708 b-i

**DOI:** 10.1038/s41550-021-01539-1

**Published:** 2022-01-13

**Authors:** David Kipping, Steve Bryson, Chris Burke, Jessie Christiansen, Kevin Hardegree-Ullman, Billy Quarles, Brad Hansen, Judit Szulágyi, Alex Teachey

**Affiliations:** 1 Department of Astronomy, Columbia University, New York, NY USA; 2 NASA Ames Research Center, Mountain View, CA USA; 3 Department of Physics and Kavli Institute for Astrophysics and Space Research, Massachusetts Institute of Technology, Cambridge, MA USA; 4 Caltech/IPAC-NASA Exoplanet Science Institute, Pasadena, CA USA; 5 Department of Physics, Astronomy, Geosciences and Engineering Technology, Valdosta State University, Valdosta, GA USA; 6 Mani Bhaumik Institute for Theoretical Physics, Department of Physics and Astronomy, UCLA, Los Angeles, CA USA; 7 Institute for Particle Physics & Astrophysics, ETH Zurich, Zürich, Switzerland; 8 Institute of Astronomy and Astrophysics, Academia Sinica, Taipei, Taiwan

**Keywords:** Exoplanets, Rings and moons

## Abstract

Exomoons represent a crucial missing puzzle piece in our efforts to understand extrasolar planetary systems. To address this deficiency, we here describe an exomoon survey of 70 cool, giant transiting exoplanet candidates found by Kepler. We identify only one exhibiting a moon-like signal that passes a battery of vetting tests: Kepler-1708 b. We show that Kepler-1708 b is a statistically validated Jupiter-sized planet orbiting a Sun-like quiescent star at 1.6 au. The signal of the exomoon candidate, Kepler-1708 b-i, is a 4.8*σ* effect and is persistent across different instrumental detrending methods, with a 1% false-positive probability via injection–recovery. Kepler-1708 b-i is ~2.6 Earth radii and is located in an approximately coplanar orbit at ~12 planetary radii from its ~1.6 au Jupiter-sized host. Future observations will be necessary to validate or reject the candidate.

## Abstract (web summary)

A 4.8*σ* exomoon candidate is found around gas giant Kepler-1708 b, which orbits at 1.6 au around its star. It is the only candidate from a dedicated survey that analysed 70 cool giant exoplanets discovered by Kepler. Kepler-1708 b-i has a radius of 2.6 Earth radii and orbits its planet at 12 planetary radii.

## Main

In the last three decades, more than 4,000 planets around stars other than the Sun, exoplanets, have been discovered. These worlds display remarkable diversity, from highly eccentric Jupiters^1^ to compact, coplanar systems of terrestrial planets^2^. In an effort to understand the formation and evolution of such systems, more detailed knowledge about their environment and properties is sought^3^—such as the existence and nature of potential satellites^4^. Given the abundance of moons in our Solar System, it is reasonable to presume that exomoons will reside around some exoplanets—which has motivated efforts to detect them^5,6^.

One of the most promising strategies for seeking exomoons focuses on transiting planets^7–9^: worlds that periodically eclipse their stars and make up the majority of the discovered exoplanets. However, the observational bias of transit surveys^10^ leads to an under-representation of long-period, cool planets—precisely the type of planet where moons are thought to be most likely due to dynamical considerations^11,12^. Nevertheless, a small sample of long-period planetary candidates was discovered by Kepler^13–17^—worlds with orbits greater than that of the Earth around the Sun. The Jupiter-sized planets amongst these are of particular interest, as satellite formation is thought to be a natural outcome of how such planets form^18^.

To date, very little is known about the prevalence and properties of exomoons. Initial surveys largely focused on planets interior to 1 au (ref. ^19^), since this was broadly the only sample available at the time. Around these relatively close-in planets, large moons appear uncommon, with the abundance of Galilean-like satellite systems measured to be <38% to 95% confidence^20^. However, amongst the longest periods of these worlds, the ~1 au Jupiter-sized planet Kepler-1625 b was reported to exhibit a timing variation and transit signature consistent with a large Neptune-sized/mass moon using Hubble Space Telescope photometry^21^. Both of these were independently recovered in one study^22^, but only one (the timing) in another^23^—shown later to be possibly due to higher systematics in their photometric reduction^24^. Much like hot Jupiters, such large moons were not widely anticipated in the literature. However, subsequent theoretical work has shown that the candidate exomoon could form through a capture scenario^25^ or a massive circumplanetary disk^26^.

With no published exomoon surveys for planets at ≳1 au, and the intriguing hint of Kepler-1625 b-i, the aforementioned Kepler sample of long-period giant planetary candidates represents one of the most promising unturned stones. To address this, we here present a survey of Kepler’s cool, gas giants.

## Results

We first curated known long-period transiting planets discovered by Kepler from the literature, selecting any object with a reported radius within a factor of two of Jupiter’s, and with either (1) a period of >400 d, (2) an equilibrium temperature of <300 K or (3) an instellation less than that of Earth (Methods). After removing any object listed as a false positive or with fewer than two available transits, our sample comprised of 73 cool giants.

After the analysis and removal of long-term systematic trends in the Kepler photometry, three targets were rejected as being of unacceptably poor quality. Light-curve detrending was performed using four different algorithms applied to two photometric reductions, with the results cross-compared and averaged (‘method marginalized’), to ensure a robust correction against algorithmic choices (Methods).

For the 70 cool giants remaining, we fitted several light-curve models to each detrended photometric time series. Using these fits, we applied a battery of initial tests to check for the presence of exomoons. These are described in detail in Methods, and include the following: (1) the Bayes factor of a photodynamical planet–moon model must be favoured over a planet-only model by at least a factor of 10 (that is, ‘strong’ evidence^27^), (2) the light curve has to be consistent with a planet on a near-circular orbit (as high eccentricities diminish the suitability for moons^28,29^) and (3) if more than two transits are available the object should exhibit transit timing variations (TTVs). For the eccentricity test, it was necessary to derive fundamental stellar properties for each host star, which was achieved through an isochrone analysis (Methods).

**Fig. 1:**
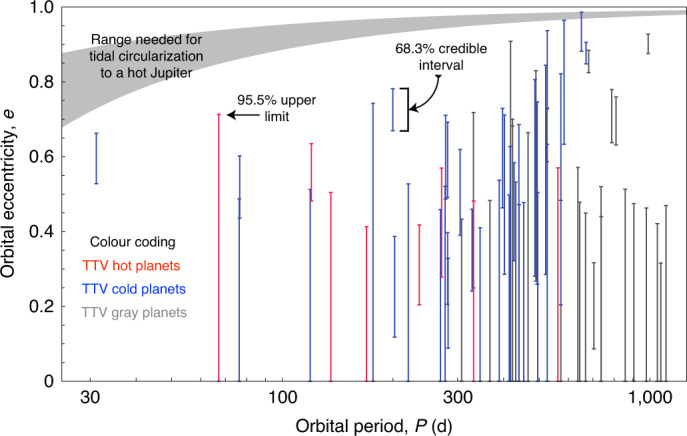
Orbital properties of the 70 cool giants. A comparison of the derived orbital eccentricities from this work (*y* axis) versus the orbital periods (previously known) for our planetary-candidate sample. We use colour (legend) to depict the dynamical ‘temperature’ via the inferred absence/presence of TTVs. Source data

**Extended Data Fig. 1:**
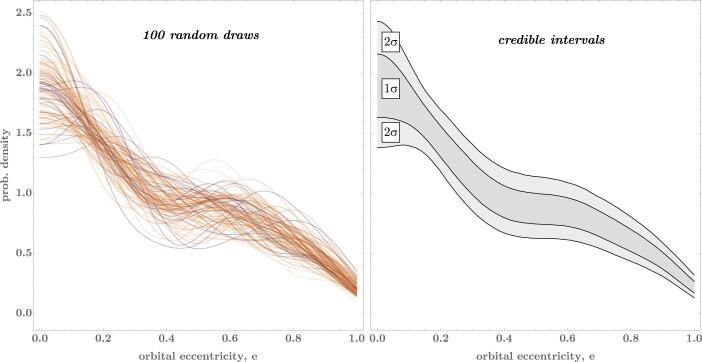
Probability distribution of the cool giant’s eccentricities. Left: We extract a random draw from the eccentricity posterior distribution of each planet and apply a smooth kernel density estimator (KDE) to the sample with a Gaussian kernel. Each line represents 1 of 100 such realisations. Right: Credible intervals evaluated using 10^5^ such samples as computed in the left panel. Source data

Although our primary goal is to search for exomoons, these tests provide some novel dynamical insights that we highlight here. First, we find no clear correlation between planetary candidates that exhibit TTVs and those with eccentric orbits (Fig. 1). Further, the eccentricity distribution does not appear sufficiently extreme to explain the origin of hot Jupiters through tidal circularization theory^30^. However, we do see tentative evidence that the eccentricity distribution consists of more than one component (Extended Data Fig. 1), indicative of multiple evolutionary paths.

From the initial trio of tests, 11 planetary candidates passed these criteria and were thus considered further (Supplementary Table 1). We emphasize that this does not mean that none of the other targets host moons; indeed, some of our moon fits may have recovered genuine moon signals. However, in each of these, there is at least one reason why the signal is weaker than that expected of a faultless detection. By rejecting these, we follow a conservative approach of only tolerating signals with zero reasons for concern.

We next applied three additional tests to the surviving 11 (Methods). Specifically, the light curves were refitted with another planet–moon model, but one that permits unphysical parameter values, such as a negative-radius moon (inverted transits) and unphysically large bulk densities for the planet and moon. This allowed us to fairly evaluate the preference of the models for (4) a non-zero moon mass, (5) a non-zero moon radius and (6) a positive moon radius.

Only three objects survive these additional checks: KIC-8681125.01^17^, KIC-5351250.06 (also known as Kepler-150 f^31^) and KIC-79068275.01^17^. At this point, we turned to more detailed vetting tailored to each object. In general, our goal is to identify if there is any basis to eliminate the objects as possible exomoons, and we work through various tests in an effort to accomplish this. As soon as a test is failed, the object is rejected from further consideration, regardless of how compelling other aspects of the exomoon-like signal may have been.

**Extended Data Fig. 2:**
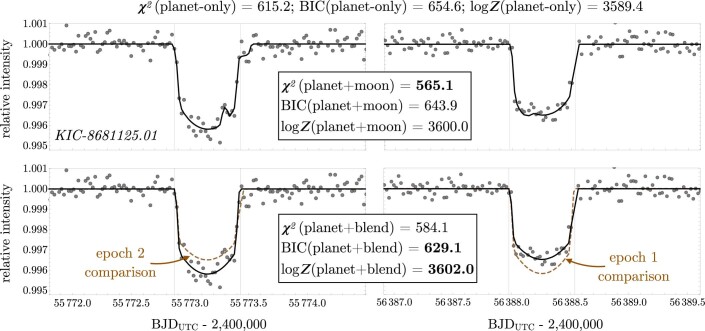
Transit light curves of KIC-8681125.01 for the first (left) and second (right) epochs. Top: Each panel shows the method marginalised detrended photometry centred on the times of transit, with the maximum likelihood planet–moon fit overlaid in solid black. Model comparison statistics are provided within the inset box. Bottom: Same as above but for a model with a single planet and variable blend factor between the two epochs. This model substantially outperforms the planet–moon model. Source data

Inspection of the best-fitting planet–moon model to KIC-8681125.01 (Extended Data Fig. 2) revealed immediate cause for scepticism. No distinct moon-like features are observed, with the signal dominated by a transit depth change between the two available epochs of $3{,}59{0}_{-130}^{+160}$ ppm and $3{,}03{0}_{-110}^{+140}$ ppm. Since the two epochs are separated by six Kepler quarters, the spacecraft has physically rolled into a distinct position, meaning that unknown background stars can contribute differently between the two epochs. No high-resolution imaging exists for this source, but Gaia lists the closest companion as 11.8 arcsec away and 1.7 mag fainter, which is somewhat too faint and distant to easily explain the required dilution. Sources interior to Gaia’s arcsecond resolving power^32^ remain possible, as (6.3 ± 0.9)% of single-planetary candidate Kepler stars have companions within 2.0 arcsec (ref. ^33^). However, as discussed in Methods, this possibility also does not easily explain the depth change, and an instrumental effect could ultimately be responsible too.

To evaluate the fitness of the blend model, we refit the light curve with the planet-only model but modifying it such that the second epoch is diluted by some factor, *γ*, from which we obtain *γ* = 1.188 ± 0.037. This model yields the highest marginal likelihood score of all models tried, implying a Bayes factor for the blend model of 6.8. Taking all these points together, questions certainly remain about the cause of the depth change, but we consider it unlikely that it is ultimately driven by an exomoon, given both the alternative possibilities and the nature of the signal.

**Extended Data Fig. 3:**
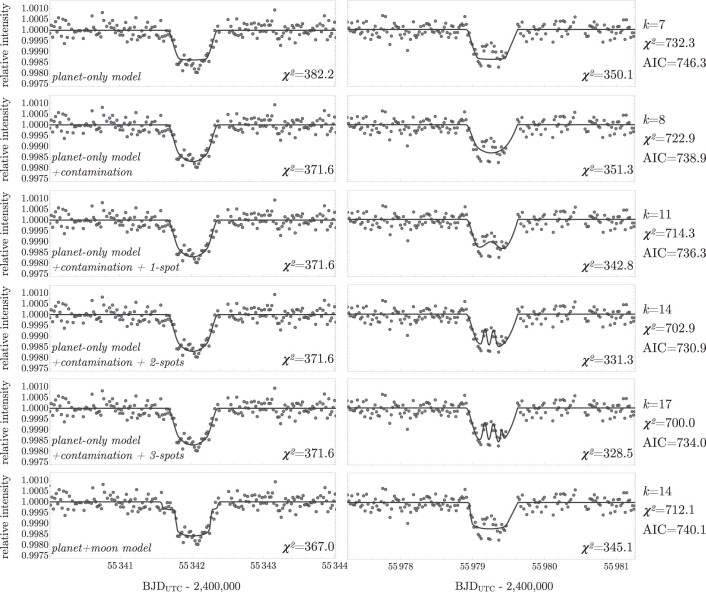
Transit light curves of KIC-5351250.06/Kepler-150f for the first (left) and second (right) epochs. Each row shows a different model fit to the same data. Whilst the planet–moon model is clearly a better fit than the planet-only model, a 2-spot model is able to out-perform either and is well motivated from the activity levels observed in the out-of-transit light curve. Source data

Turning to Kepler-150 f, inspection of the planet–moon fit reveals in-transit morphological differences between the two available epochs. Unlike KIC-8681125.01, this is not characterized by a simple depth change, but rather complex morphology, particularly within the second event (Extended Data Fig. 3). This raises concern that the signal is spurious and caused by Kepler-150 f passing over dark starspots on the stellar surface, as has been reported previously^34^.

As the star rotates, spots cause the brightness to periodically change. If the second transit was afflicted by spots, one might expect it to coincide with a flux minimum in these rotational modulations^34^, when we are observing the spot-covered face of the star—and this is indeed the case here. Further, the star is known to be rotationally active with a reported^35^ periodicity of 17.6 d and amplitude of 10.9 mmag. This far exceeds the depth of Kepler-150 f’s transit (~1.5 mmag), indicating that the spot-covered area of the star is much larger than the planet itself and thus large transit distortions can occur. Finally, we show in Methods that a modification to the planet-only model that includes two spot crossings (using a simplified prescription) yields a Δ*χ*^2^ = 9.2 improved fit to the transits versus the planet–moon model, despite using the same number of free parameters. On this basis, we conclude that this is most probably spot-driven activity rather than an exomoon signature.

**Fig. 2:**
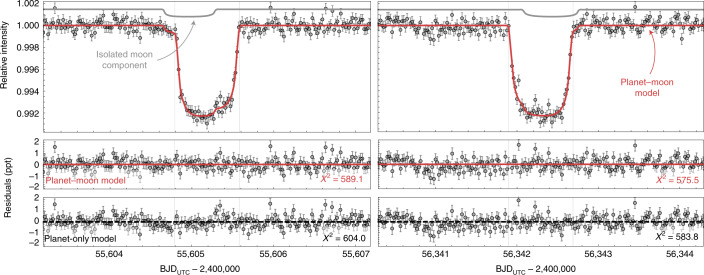
Transit light curves of Kepler-1708 b. The left/right column shows the first/second transit epoch, with the maximum-likelihood planet–moon model overlaid in solid red. The grey line above shows the contribution of the moon in isolation. Lower panels show the residuals between the planet–moon model and the data, as well as the planet-only model. BJD, barycentric Julian date; UTC, coordinated universal time. Source data

Finally, we turn to KIC-7906827.01. As with the other two, only two transits were available given the long period of *P* = 737.1 d. The Bayes factor of the planet–moon model against the planet only is 11.9, formally passing our threshold of 10 (strong evidence on the Kass and Raftery scale^27^). Inspection of the maximum-likelihood moon fit, shown in Fig. 2, reveals that the signal is driven by an unexpected decrease in brightness on the shoulder of preceding the first planetary transit, as well as a corresponding increase in brightness preceding the egress of that same event. The time interval between these two anomalies is approximately equal to the duration of the planetary transit, which is consistent with that expected for an exomoon^36^. The second transit shows more marginal evidence for a similar effect. The planet–moon model is able to well explain these features, indicative of an exomoon on a fairly compact orbit, to explain the close proximity of the anomalies to the main transit. In a raw *χ*^2^ sense, the inclusion of the exomoon leads to a Δ*χ*^2^ = 23.2 improved fit, indicating a 4.8*σ* effect. This does not penalize the model for its extra complexity, but that is accounted for in the previously mentioned Bayes factor calculation of 11.9.

**Extended Data Fig. 4:**
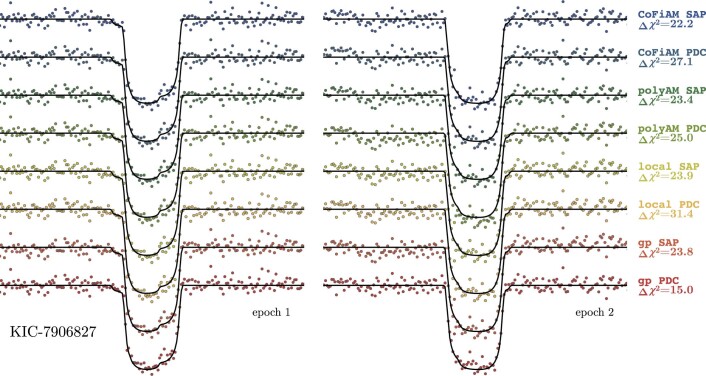
Detrended transit light curves of KIC-7906827.01 for the first (left) and second (right) epochs. Each row shows a different combination of light curve detrending method and input data, which are combined to build the method marginalised product. For each, we overlay the maximum *a posteriori* planet–moon model as conditioned upon the method marginalised light curve, and a comparison of how much better it matches the data versus the planet-only model, in a chi-squared sense. Source data

Our first concern was whether these undulations could be a spurious product of the detrending process. Inspection of the individual-method-based detrendings, rather than the method marginalization, shows that the anomalies are present in all detrendings (Extended Data Fig. 4), and further the planet–moon fit from the method-marginalized light curve is always a closer match than the planet-only fit (Methods). Thus, the moon-like signal appears robust against detrending choices.

Unlike KIC-8682235.01, transit 1’s pre-ingress dip cannot be a starspot crossing, since it occurs before the planet even enters the stellar disk. It is also unlikely to be caused by an unseen contaminant star—this would require such a star to coincidentally undergo a transit at almost precisely the same instant as the unrelated source star (although we investigate this possibility shortly). Additionally, unlike KIC-8682235, the two epochs are separated by an integer number of four quarters, from Q8 to Q16, thus placing the star on the same detector module in each quarter, and indeed the same optimal aperture is used. Thus, any difference between the two epochs cannot be caused by a contaminant being present in one epoch but not the other.

**Extended Data Fig. 5:**
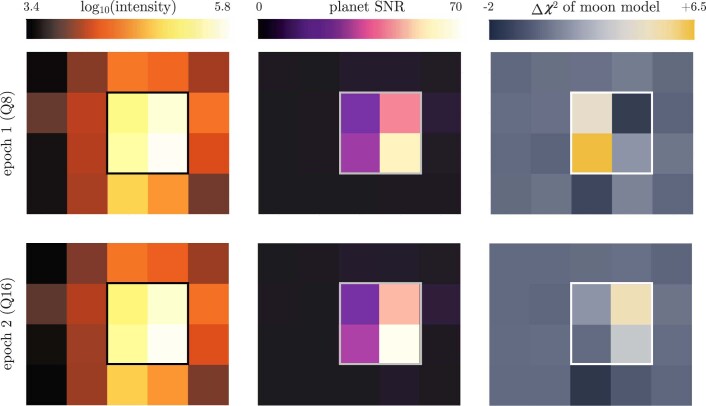
Pixel-level comparison the two transits of KIC-7906827.01. Left: Pixel log-intensity is shown for the postage stamp downloaded for KIC-7906827 from the *Kepler* spacecraft, for epochs 1 (top) and 2 (bottom). The black solid outline shows the optimal aperture selected by the *Kepler* pipeline. Middle: Same as the left, except we show the signal to noise ratio (SNR) of the planetary transit signal in each pixel. As expected, the transit signal is colocated with the brightest source in view. Right: Same as the middle, except we show the chi-squared improvement of the planet–moon model over the planet-only model in each pixel light curve after local detrending. As expected, the moon signal appears colocated with the target. Source data

Detailed inspection of the pixel light curves shows that the pixels of highest planetary transit signal-to-noise ratio (SNR) coincide with the highest-flux region, as expected. We also applied this to the pixel location of the moon signature, by evaluating the Δ*χ*^2^ between the planet–moon and planet-only models in each pixel, with local detrending of the pixel light curves and normalization. This test was used for Kepler-90 g in 2014, indicating that a hypothesized exomoon was a false positive^37^, possibly caused by a sudden pixel sensitivity dropout event^38^. In contrast, we find here (Methods) that the candidate moon’s SNR is collocated with the planetary signal, consistent with a genuine signal (Extended Data Fig. 5).

Analysis of the flux-weighted centroids reveals a small shift of {−0.52 ± 0.06, +0.62 ± 0.05} millipixels in the {*X*, *Y*} directions during the two transits of KIC-7906827.01. This can sometimes indicate that the transit occurs on a different star than assumed, potentially ruling out KIC-7906827.01 as a bona fide planet, but it can also simply occur because of nearby stars within the aperture^39^. A detailed centroid analysis (Methods) shows that amongst the known nearby stars in the Gaia catalogue only KIC-7906827 could plausibly be the host of the transit signal. The shift is indeed broadly consistent with that expected as a result of the known stars, and the estimated blend probability was calculated to be 2.6 × 10^−6^.

Although the centroids indicate that the signal is a real planet, other information (such as the transit light-curve shape) can also be used to assess this hypothesis. Accordingly, we used the vespa package^40^ to rigorously calculate a statistical probability of planethood (Methods). From this, we estimate the false-positive probability (FPP) to be 0.024%, substantially below the 1% threshold typically used to define a ‘validated’ exoplanet^40^—we thus refer to the planet as Kepler-1708 b henceforth.

With Kepler-1708 b validated, we return to the exomoon signal. A formal assumption in the light-curve fits is that the noise is described by an independent normal distribution. Time-correlated noise would render this assumption invalid and could introduce deviations into the photometry to such a degree that the planet–moon model fits are spuriously favoured—a false positive. The act of detrending the photometry attenuates this possibility, but residual correlated noise could still persist. Although we see no evidence for this (Methods), it cannot be excluded and to some degree will always be present in real-world conditions. We thus performed an injection–recovery exercise, where we injected the planet-only template for Kepler-1708 b into the KIC-7906827 photometry at random times away from the real events and performed the same battery of tests to see how often we would erroneously claim an exomoon. By using real light curves, any time-correlated noise structure associated with the source is properly accounted for.

**Fig. 3:**
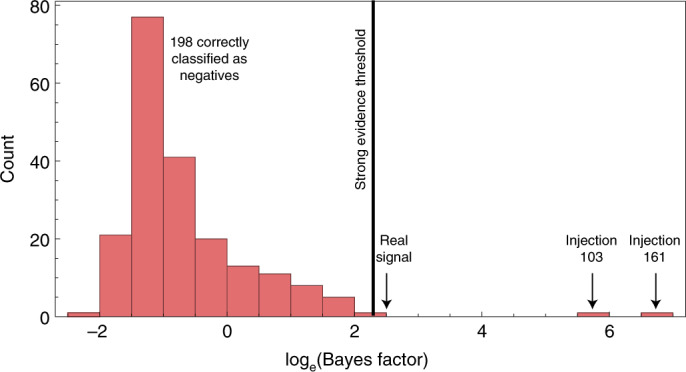
FPP calculation for Kepler-1708 b-i. Histogram of the log Bayes factor between a planet–moon and a planet-only model from 200 fake planet-only signals injected into the light curve. Two signals pass the threshold (=log_e_10) and have positive radii, indicating a 1% FPP. Source data

Computational constraints limit us to 200 such fake systems; amongst these injections we find two cases where we would spuriously claim an exomoon (Methods), and thus the FPP for the exomoon signal is $1.{0}_{-1.0}^{+0.7}$%. If the signal is indeed not from time-correlated noise, the most likely astrophysical false positive is an unseen second transiting planet, for which we find the probability is ≲1% (Fig. 3).

## Discussion

From a survey of 70 cool giant exoplanets, we find no compelling evidence for an exomoon around any, bar one: KIC-7906827.01/Kepler-1708 b. This candidate is currently uncertain, with an estimated FPP of being an astrophysical signal of ~1% and a ≲1% probability of a previously undetected transiting planet causing such a signal. One detection from a sample of 70 and a 1% FPP naively appears consistent with zero moons, but it is also fully consistent with one real signal, with the actual odds being dependent upon the underlying (and unknown) occurrence rate of large exomoons (Methods). In short, we can find no grounds to reject Kepler-1708 b-i as an exomoon candidate at this time, but urge both caution and further observations.

Our photodynamical model predicts a planetary mass of <4.6 *M*_J_ (2*σ*), corresponding to a predicted radial-velocity amplitude of <98 m s^−1^. As a faint source Kepler bandpass ((*K*_P_) = 15.8), radial-velocity detection would be a major challenge. TTVs are generally expected and could be observed with future transits. Although we only have photodynamically derived upper limits on the planet and satellite masses, we can forecast on the basis of their radii^41^ that the TTV amplitude has a 95% confidence range between 1.2 and 77.0 min. Future observations with the Hubble Space Telescope, James Webb Space Telescope or PLATO could seek these TTVs or repeated moon transits (≃500 ppm).

Kepler-1708 b-i joins Kepler-1625 b-i^42^ as another example of an unexpectedly large exomoon candidate—echoing the surprise that hot-Jupiter discoveries elicited in the mid-1990s^43^. The basic properties are listed in Table 1, and can be summarized as those of a mini-Neptune moon orbiting approximately 12 planetary radii around a Jupiter-sized planet, which itself orbits a Sun-like star at 1.6 au. Compared with Kepler-1625 b-i, the moon candidate is substantially smaller, on a tighter orbit and more consistent with a coplanar geometry. Although the reality of Kepler-1625 b-i remains unclear^44^, the existence of this second candidate challenges us to consider the origins of such large moons.

**Table 1:** System parameters for Kepler-1708 b

Parameter	Description	Value
*R*_P_/*R*_⋆_	Planet–star radius ratio	$0.081{8}_{-0.0010}^{+0.0011}$
*P*_P_ (d)	Planet’s orbital period	$737.113{1}_{-0.0077}^{+0.00146}$
*b*_P_	Impact parameter of the planet	<0.37 (2*σ*)
*a*_P_/*R*_⋆_	Planet–star radius ratio	$317.{9}_{-8.4}^{+5.2}$
*R*_P_ (*R*_⊕_)	Planetary radius	$9.9{6}_{-0.59}^{+0.60}$
*M*_P_ (*M*_J_)	Planetary mass	<4.6 (2*σ*)
*a*_P_ (au)	Planet’s orbital radius	$1.6{4}_{-0.10}^{+0.10}$
*e*_P_	Orbital eccentricity of the planet	<0.40 (2*σ*)
*S*_P_ (=*S*_S_) (*S*_⊕_)	Instellation cf. Earth	$0.56{1}_{-0.068}^{+0.074}$
*P*_S_ (d)	Satellite’s orbital period	$4.{6}_{-1.8}^{+3.1}$
*a*_SP_/*R*_P_	Satellite’s orbital radius in planetary radii	$11.{7}_{-2.2}^{+3.9}$
*i*_S_ (°)	Satellite’s inclination cf. planetary orbit	${9}_{-45}^{+38}$
*Ω*_S_ (°)	Satellite’s longitude of the ascending node	${6}_{-150}^{+140}$
*R*_S_/*R*_P_	Satellite–planet radius ratio	$0.26{3}_{-0.042}^{+0.040}$
*M*_S_/*M*_P_	Satellite–planet mass ratio	<0.11 (2*σ*)
TTV (min)	Predicted TTV amplitude	<41 (2*σ*)
*R*_S_ (*R*_⊕_)	Satellite radius	$2.6{1}_{-0.43}^{+0.42}$
*M*_S_ (*M*_⊕_)	Satellite mass	<37 (2*σ*)

We first consider the moon’s possible orbital migration from tidal interactions with Kepler-1708 b. We evolved a constant-time-lag tidal model^45^ using the system parameters from our posterior samples^46^. The tidal model adopts Jupiter-like parameters for the tidal Love number *k*_2J_ (ref. ^47^), moment of inertia^47^ and time lag^48^. We evolved the models over 10 Gyr (≃99.5% confidence upper limit on stellar age), assuming that the moon forms in situ at twice the Roche limit and that the planet has a initial spin period of 5–10 h. Over this timescale, the moon begins well beyond the corotation radius and thus slowly migrates outwards. Over the full 10 Gyr simulation, the moon migrates to ~20 *R*_P_, which is both well within the Hill stability limit (~250 *R*_P_) and consistent with our favoured solution of ~12 *R*_P_.

The fact that this candidate can plausibly migrate outwards via tides blurs the distinction between formation scenarios, as any model that produces a massive moon on a compact orbit can match the observations. There are several broad scenarios for moon formation: planet–planet collisions, formation of moons within gaseous circumplanetary disks (for example the Galilean moons) or direct capture—either by tidal dissipation or pulldown during the growth of the planet. For a gaseous planet, the first scenario is unlikely to produce a debris disk massive enough to form a moon this large. The moon is also at the extreme end of the mass range produced by primordial disks in the traditional core-collapse picture of giant-planet formation^49–51^, but is easier in the case where planets form by disk instability^49,52^. Such models also naturally produce moons on low-inclination orbits. Direct capture by tidal dissipation is also possible, although the parameter range for capture without merger is limited. Pulldown capture can produce large moons within ~10 Jupiter radii, with a wide range of inclinations depending on the timescale for planetary growth. Low inclinations such as those observed here argue for a slower envelope growth^25^.

Together then, the formation and properties of a moon such as this certainly challenge conventional thinking, but plausible mechanisms have been previously proposed. Ultimately, the reality of supermoons such as Kepler-1708 b-i and Kepler-1625 b-i will require follow-up transit photometry, as both their nature and supporting evidence demand appropriate scepticism at this time.

## Methods

### Target selection

The focus of this work is the population of cool, giant transiting exoplanets observed by Kepler. The most comprehensive catalogue of Kepler transiting planet candidates comes from the NASA Exoplanet Archive (NEA^53^) and thus we began by downloading this catalogue at the start of this investigation (27 March 2018). Not all Kepler objects of interest in the catalogue are viable planet candidates though, and so we applied a cut to remove any objects that have been dispositioned as likely false positives by the NEA.

Both the terms ‘cool’ and ‘giant’ are somewhat subjective and thus require a clear definition for the purposes of target selection. For giant, we elected to use a cut of $\hat{{R}_{\mathrm{P}}} > 0.5$ *R*_J_, where $\hat{{R}_{\mathrm{P}}}$ is the most probable radius value listed in the NEA. This choice is motivated to minimize the number of sub-Neptunes that make it into our sample, and thus focus on Jupiter-sized worlds.

The definition of cool is again subjective, but here we are primarily interested in planets in environments cooler than that of the Earth. Using a simple instellation or temperature cut alone is inadequate though, as these values depend upon the stellar parameters, which have been subject to substantial revision over time^54^. On this basis, they may not be reliable in isolation as a means of capturing all of the cool giants. Instead, we apply three different definitions of cool and accept giant planets that satisfy any of the three. These criteria are $\hat{{S}_{\mathrm{P}}} < 1$ *S*_⊕_, $\hat{{T}_{\mathrm{P}}} < 300$ K or $\hat{{P}_{\mathrm{P}}} > 400$ d—where *T*_P_ is the blackbody equilibrium temperature of the planet.

Using these cuts, 48 Kepler objects of interest were identified from the NEA. However, we noted that a subset of these had suspiciously large radii. We thus applied an additional cut to remove any with best reported planetary radii in excess of 2 Jupiter radii (removing KIC-3644071.01, KIC-6426592.01, KIC-6443093.01, KIC-9025662.01, KIC-9011955.01, KIC-8240617.01 and KIC-8868364.01). This left us with 41 unique planetary candidates. Of particular note is KIC-5437945, which possesses two cool giants associated with a single source.

At this point, we introduce another cut: that at least two transits have been observed by Kepler. Without the two transits, the orbital period cannot be precisely measured and this in turn makes it impossible to measure the eccentricity of the planets photometrically—a test we will depend on later in our exomoon analysis. Of the 41 cool giants from the NEA, 5 were found to only exhibit a single transit in the available Kepler data (KIC-2162635.01, KIC-3230491.01, KIC-3962440.01, KIC-11342550.01 and KIC-11709124.02) and thus were excluded. This leaves us with 36 cool giants.

Although the NEA is the most complete catalogue available, long-period planets are more challenging to find than their shorter-period counterparts, and thus numerous independent studies have identified long-period exoplanetary candidates that were not present in the NEA. In particular, we identified several additional studies^13–16^, which we inspected in an effort to locate any additional possible planets missed thus far. In what follows, we attempt to apply the same filters as before, such as exhibiting at least two transits, but note that in some cases the radius and instellation values had not been computed by the original authors.

From the work of Wang and colleagues^13^, KIC-8012732, KIC-9413313 and KIC-11465813 were flagged as exhibiting three or four visible transits and have long periods (431 d, 440 d and 671 d) and large radii (9.8 *R*_J_ and 13.8 *R*_J_). KIC-5437945 and KIC-7619236 also satisfy the criteria but are already included in the NEA catalogue. KIC-5652983 is long period and large but has been argued to be a likely false positive^13^ due to the observation of large radial-velocity variations. Amongst the two-transit cases, KIC-5732155, KIC-6191521 and KIC-10255705 are also added, which leads to six new cool giants from this sample.

From the work of Uehara and colleagues^14^, only KIC-10460629 exhibits at least two transits, separated by 525 d. Although the radius is not computed, the grazing, deep (2%) transit is plausibly a giant around a diminutive star and thus was included in what follows.

From the work of Foreman-Mackey and colleagues^15^, only three of the candidates have more than two transits: KIC-3239945, KIC-8410697 and KIC-8800954. However, KIC-3239945 is already found in our NEA catalogue and KIC-8800954 has a radius of 0.39 *R*_J_, making it too small for our sample. Accordingly, we only add KIC-8410697, of period 1,047 d and 0.70 *R*_J_.

From the work of Wheeler and Kipping^16^, we pick an extra transiter of 3% depth but unreported radius (KIC-8508736) with a period of 681 d.

Combining these targets with the NEA sample yielded 45 cool giants.

As our study progressed, spanning multiple years, the possibility of new cool-giant detections appearing in the NEA grew. To address this, we re-ran our NEA search on 12 June 2019, which identified 24 new cool giants. Of these, six were single-transit systems (KIC-2162635, KIC-3230491, KIC-3644071, KIC-3962440, KIC-11342550 and KIC-11709142) and were thus removed. In addition, upon querying KIC-8308347 in the Mikulski Archive for Space Telescopes, we noted that it had been flagged as an ‘eclipsing binary: likely false positive’ under condition flag and thus rejected it in what follows. These new inclusions raise our total number of cool giants to 62.

As a final addition to our catalogue, a recently published study^17^ (although quite far into our own analysis) presented 23 long-period Kepler planetary candidates. Of these, 14 were suitable for our study and all but one were double-transit systems. However, three of these were already included in our sample by this point (KIC-3756801, KIC-9663113 and KIC-10460629), meaning that the ref. ^17^ sample added 11 cool giants to our ensemble. Of the new objects, KIC-5351250 is of particular note since it represents the fifth planetary candidate in the Kepler-150 system^31^. Together, this brings our final catalogue of cool giants up to 73 planetary candidates. Three of these were found to exhibit unacceptably high correlated noise structure in the light curves and were thus rejected, as described later in this section. The remaining 70 are listed in Supplementary Table 1.

### Data preparation

For each target, the light-curve files were downloaded from the Mikulski Archive for Space Telescopes, using primarily the long-cadence data but short cadence where available. However, given the long-period nature of our transiting bodies, the value of short cadence is considerably less than that of typical transiters^55^. In all cases, the data were processed as part of the 25th and final data release issued by the Kepler science team^56^, dubbed DR25 hereafter.

For all light curves, we trimmed any points with an error flag equal to anything other than zero—thus removing points known to be afflicted by effects such as reaction-wheel zero crossings^57^. Additional outliers (for example unidentified cosmic-ray hits^58^) were removed independently for the ‘simple aperture photometry’ (SAP) and ‘pre-search data conditioning’ (PDC^59^) light curves, by flagging points more than 3*σ* deviant from a moving median of bandwidth 20 long cadences.

Kepler light curves exhibit modulations in intensity due to myriad effects. Ultimately, the short-term modulations corresponding to a transit are of central interest to this study, but longer-term variability is also present and introduces sizable trends that require correction. Such variability could originate from the instrument (for example focus drift^60^) or the parent star (for example rotational modulations^61^). In what follows, we describe our approach for detrending these effects.

As a brief aside, we note that short-term variability on the same timescale as the transits can also be present (for example pulsations in evolved stars^62^) and is generally much more difficult to remove, since it is not separable in the frequency domain. Consequently, attempts to remove such noise come at grave risk of distorting a transit signal of interest. Given that our primary objective is to look for exomoons, which manifest as small undulations on this timescale, it was considered an unacceptable risk to attempt to remove such short-term variability—since such efforts may in fact introduce false positives into the time series. Instead, the philosophy in what follows is to use statistical tests to identify light curves corrupted by such noise and simply discard them. This naturally comes at the expense of increasing our false-negative rate, since such systems are not even analysed further.

For each cool-giant target, we detrend the light curves of the individual transit epochs individually, rather than imposing the idea that the noise in one quarter need be representative of others. This is largely motivated by the fact that the spacecraft rolls every quarter and thus sources appear on different silicon with different optimal apertures, blend contaminations and CCD (charge-coupled device) behaviours. In addition, we adopt the approach of detrending each transit epoch in eight different ways. The reasoning here is that, although we generally consider each of the eight different methods to be fairly accurate (else we would not be using them), we cannot guarantee that any of them are going to work in every situation. From experience, peculiarities in particular light curves can interact with detrending algorithms in unanticipated ways, leading to anything from a complete failure to a subtle residual trend. Fundamentally, any claim we make about the presence of an exomoon needs to be robust against choices made in this detrending stage, and a path to achieving this is to simply use multiple detrending methods and compare.

The details of the different detrending algorithms used are presented shortly, but once in hand they are combined into a single data product (per transit epoch) known as a method-marginalized light curve^21^. In this work, we generate such light curves by simply taking the median of the multiple detrended intensities at each time stamp. The formal uncertainty on each photometric data point is also inflated by adding it in quadrature to 1.4286 multiplied by the median absolute deviation between the methods. Median statistics are used throughout to mitigate the influence of failed detrending(s). In this way, we increase the robustness of our light products against detrending choices and also inflate the errors to propagate the uncertainty in the detrending procedure itself.

As an additional safeguard against poorly detrended light curves, we compute two light-curve statistics to measure their Gaussianity. If any of the eight light curves fail this test, they are rejected before the method-marginalization procedure. For the first test, we bin the light curves (after removing the transits) into ever larger bins and compute the s.d. versus bin size, against which we then fit a linear slope in log–log space. For such a plot, the slope should be minus one-half, reflecting the behaviour of Poisson counting of independent measures. However, time-correlated noise structure will lead to a shallower slope that can be used to flag such problematic sources^63^. We thus generate 1,000 light curves of precisely the same time sampling and pure Gaussian noise and measure their slopes in this way. This allows us to construct a distribution of expected slope values. If the real slope deviates from the Monte Carlo experiments with a *P* value exceeding 2*σ*, the light curve is flagged as non-Gaussian.

For the second test, we compute the Durbin–Watson^64^ statistic of the unbinned light curves (after removing the transits). This is essentially a test for autocorrelation at the timescale of the data’s cadence, where uncorrelated time series should yield a score of 2. As before, we test for non-Gaussian cases by generating 1,000 fake Gaussian light curves at the same time sampling and scoring their Durbin–Watson metrics. If the real light curve is deviant from this distribution by more than 2*σ*, the light curve is rejected.

The above describes how we combine eight light curves detrended independently, but we have yet to describe how these eight light curves are generated in the first place—to which we turn in what follows. In total, four different detrending algorithms are used, which are then applied to the SAP and PDC data to give eight light curves. The four algorithms are described now.

CoFiAM. Cosine Filtering with Autocorrelation Minimization (CoFiAM) builds upon the cosine filtering approach previously developed for CoRoT^65^ data. Cosine filtering is attractive because it behaves in a predictable manner in the frequency domain, unlike the other methods used here, which leak power across frequency space. Fourier decomposition of the transit morphology reveals dominant power at the timescale of the transit duration and higher frequencies^66^. Thus, by only removing frequencies substantially lower than this, we can ensure that the morphology of the transit is not distorted by the detrending process itself. On the other hand, cosine filtering is problematic in that we could regress a very large number of cosines to the data. Much like fitting high-order polynomials, predictions from such model become unstable at high order. In our case, we train on the out-of-transit data (in fact the entire quarter) and interpolate the model into the transit window, thus introducing the possibility of high-order instabilities here.

This is where our implementation deviates from that used for CoRoT^65^, to account for this effect. We detrend the light curve in up to 30 different ways, in each case choosing a different number of cosine components to include. The simplest model is a single cosine of frequency given by twice the baseline of available observations (thus looking like a quadratic trend)—known as the basic frequency. At each step, we add another cosine term of higher frequency to the function (equal to a harmonic of the basic frequency), train the updated model, detrend the light curve and compute statistics concerning the quality of the detrending. We continue up to 30 harmonics, or until we hit 1.5 times the reported transit duration. From the 30 options, we pick the one that leads to the most uncorrelated light curve—as measured from the Durbin–Watson statistic evaluated on the data surrounding (but not including) the transit (specifically to within six transit durations either side). These local data are then exported with the data further away from transit trimmed at this point. We direct the reader to our previous work^67^ for more details of this approach, including the underlying formulae used.

PolyAM. Polynomial Detrending with Autocorrelation Minimization (PolyAM) is similar to the above except that the basis function is changed from a series of harmonic cosines to polynomials. As before 30 different possible maximum polynomial orders are attempted from 1st to 30th order, and as before for each epoch the least autocorrelated light curve is selected as the accepted detrending on a transit-by-transit basis.

local. The next approach again uses polynomials, and again up to 30th order, but this time the final accepted polynomial order is that which leads to the lowest Bayesian information criterion (BIC)^68^ as computed on the data directly surrounding the transit (specifically to within six transit durations). This is arguably the simplest of the four algorithms attempted and is a fairly typical strategy in the analysis of short-period transiters^69^.

GP. Finally, we implemented a Gaussian process (GP) regression to the light curve. As with all of the methods above, the transits are masked during the regression by using the best available ephemeris. We implemented the regression using a squared exponential kernel where the hyperparameters (for example length scale) are optimized for each epoch independently. For consistency, we only export the data that is within six transit durations of the transit, although technically the entire segment (±0.5 orbital periods of each transit) is detrended.

As a final note, recall that during the method-marginalization process we perform checks to identify detrended light curves that do not conform to Gaussian behaviour. If all eight detrendings of a given epoch fail these tests, then there will be no accepted light curves to combine and thus the epoch is dropped. In some cases, this can reduce the number of available transits (after detrending) to fewer than two—thereby making it fail our basic criteria of presenting two epochs or more. We found that this happened for three objects in our sample, KIC-10255705, KIC-11513366 and KIC-6309307. This removal of these three targets reduces our sample size from 73 to 70 (which are the ones listed in Supplementary Table 1).

### Isochrone analysis

The 70 transiting planet candidates are associated with distinct stars from one another, with the exception of one pair associated with KIC-5437945, leading to 69 unique stars. To derive physical dimensions for the planetary candidates, it is necessary to first derive stellar parameters. This is accomplished using an isochrone analysis, which compares observable quantities associated with a star against a grid of stellar models, assuming different masses, radii, ages and so on. In this way, best-matching solutions can be inferred in a Bayesian framework to derive posterior distributions for the stellar properties.

Given that our stars are broadly FGK type, we elected to use the Dartmouth stellar isochrone models^70^ to describe these stars. For each target, we took the Gaia DR2 parallax^71^, the Kepler bandpass apparent magnitude, and the stellar atmosphere properties reported in the Kepler DR25 catalogue^54^, and appended them to a star.ini file along with their associated errors. These were then fed into the isochrones package^72^ to obtain a posteriori fundamental stellar parameters, including the mean density of the host star (*ρ*_⋆_). These fundamental parameters are reported in Supplementary Table 2 and were used later in our analysis for deriving planet/moon radii/masses.

Due to the particular interest of the target KIC-7906827, we updated our isochrone analysis to include the Gaia DR3 parallax when it became available (reducing the parallax uncertainty by 25%). We also elected to use the stellar atmosphere properties from the transit detection work^17^ (${T}_{{{{\rm{eff}}}}}=6{,}15{7}_{-202}^{+231}$ K, ${{\mathrm{log}}}\,g=4.3{7}_{-0.05}^{+0.04}$, Fe/H $=0.{0}_{-0.2}^{+0.2}$) rather than the DR25 catalogue^54^ (*T*_eff_ = 5,977 ± 176 K, log *g* = 4.39 ± 0.12, Fe/H = −0.08 ± 0.26), although we note that these values are clearly very similar. The system parameters listed in Table 1 correspond to these revised choices, for which the associated fundamental stellar parameters are *M*_⋆_ = 1.088 ± 0.072 *M*_⊙_, *R*_⋆_ = 1.117 ± 0.064 *R*_⊙_, log_10_[*A* (yr)] = 9.50 ± 0.31, log_10_[*L*_⋆_ (*L*_☉_)] = 0.182 ± 0.082, *d* = 1,712 ± 75 pc and log_10_[*ρ*_⋆_ (g cm^−3^)] = 0.042 ± 0.065.

We note that these are not the same fundamental stellar parameters for KIC-7906827 as listed in Supplementary Table 2, which originate from the original Kepler DR25 and Gaia DR2 inputs. Once again though, we note that there is very little difference between the two, with *M*_⋆_ = 1.056 ± 0.067 *M*_⊙_, *R*_⋆_ = 1.098 ± 0.095 *R*_⊙_, log_10_[*A* (yr)] = 9.61 ± 0.33, log_10_[*L*_⋆_ (*L*_☉_)] = 0.140 ± 0.087, *d* = 1,750 ± 100 pc and log_10_[*ρ*_⋆_ (g cm^−3^)] = 0.05 ± 0.11. As a final point of comparison, both sets of values are in good agreement with the independent analysis (using Gaia DR2) by Berger and colleagues^73^, who find ${M}_{\star }=1.06{1}_{-0.079}^{+0.073}$ *M*_⊙_, ${R}_{\star }=1.14{1}_{-0.066}^{+0.073}$ *R*_⊙_, log_10_[*L*_⋆_ (*L*_☉_)] = 0.140 ± 0.087 and *d* = 1,640 ± 100 pc.

### Light-curve fits

For planets exhibiting three or more transits, at least three different light-curve models, or hypotheses, were proposed to explain the data. The first is model ${{{\mathcal{P}}}}$, which represents the null hypothesis of a transiting planet orbiting its star on a strictly Keplerian orbit. In this case, the Mandel–Agol^74^ light-curve algorithm is used. The second hypothesis, model ${{{\mathcal{T}}}}$, expands upon the first by adding TTVs. This is formally accounted for using same algorithm again but allowing each transit epoch to have a unique time of transit minimum, *τ*_*i*_. The third model considered is that of a planet–moon system, ${{{\mathcal{M}}}}$, generated using the LUNA photodynamic algorithm^36^. For planets exhibiting just two transits, TTVs cannot be distinguished from a linear ephemeris and thus model ${{{\mathcal{T}}}}$ was not used.

In each model, the limb darkening of the star is modelled with a quadratic limb darkening law using the *q*_1_–*q*_2_ reparameterization^75^. Since the majority of light curves are long cadence, the potentially compelling light-curve smearing effect is accounted for by employing the numerical resampling method^55^ (with *N*_resamp._ = 30). Finally, contaminated light from nearby sources is tabulated in the Kepler fits files as ‘CROWDSAP’ and this value is used in a blend correction to each quarter’s light curve using a previously published method^76^. We also note that the models formally assume circular orbits, although elliptical planets are almost perfectly described by these models too^77^, with the exception that the fitted stellar density will be skewed away from the true value^78^. Exomoon orbits are also treated as circular, which is justified on the basis of the expected rapid circularization timescales^79^.

Regressions were executed using the multimodal nested sampling algorithm MultiNest^80^ with 4,000 live points. The advantage of using MultiNest over conventional Markov chain Monte Carlo (MCMC) methods is the ability to sample disparate modes and efficiently evaluate the marginal likelihood of the proposed hypotheses, which is used later in Bayesian model selection.

For model ${{{\mathcal{P}}}}$, seven parameters fully describe the light-curve model and thus are the free parameters in these fits. These are (1) *P*, (2) *τ*, (3) *p*, the ratio of radii between the planet and the star, (4) *b*, (5) *ρ*_⋆_, (6) *q*_1_, the first limb-darkening coefficient, and (7) *q*_2_, the second limb-darkening coefficient. Uniform priors are adopted for all except the light-curve-derived stellar density (under the assumption of a circular orbit), *ρ*_⋆,circ_, for which we use a log-uniform prior between 10^−3^ g cm^−3^ and 10^3^ g cm^−3^.

For model ${{{\mathcal{T}}}}$, we have 5 + *N* parameters, where *N* is the number of available transit epochs. The first five terms are the same as those of model ${{{\mathcal{P}}}}$ except for *P* and *τ*. The extra *N* terms are the individual times of transit minimum for each epoch.

Finally, for model ${{{\mathcal{M}}}}$ we have 14 free parameters. The first seven are simply the same as for model ${{{\mathcal{P}}}}$ but the last seven describe the exomoon. Specifically, these are (1) *P*_S_, (2) *a*_SP_/*R*_P_, (3) *R*_SP_, the ratio of radii between the satellite and the planet, (4) *M*_SP_, the ratio of masses between the satellite and the planet, (5) *ϕ*_S_, the orbital phase of the satellite at the instant of planet–star inferior conjunction during the reference epoch, (6) cos(*i*_S_) and (7) *Ω*_S_. As before, uniform priors are adopted for all with the exception of *P*_S_, which uses a log-uniform prior from 75 min to the period corresponding to one Hill radius. The semimajor axis of the satellite has a uniform prior from 2 to 100 planetary radii.

For all models, a normal likelihood function is adopted. The only addition made to this is that we applied a likelihood penalty to model ${{{\mathcal{M}}}}$, which explored unphysical parameter combinations. Specifically, we calculate the satellite and planet density using previously published expressions^81^ and reject any samples for which *ρ*_S_ > 20 g cm^−3^ or *ρ*_P_ > 150 g cm^−3^ or *ρ*_P_ < 0.5 g cm^−3^, in an effort to keep the sampler in the region of physically plausible solutions. Additionally, solutions where the satellite period exceeds 93.09% of the Hill sphere are unstable even for retrograde orbits^28^, and are thus rejected.

### Initial checks for exomoon candidacy

The primary objective of this work is to search for new possible exomoon candidates amongst Kepler’s cool-giant sample. One of the first observable effects predicted for exomoons originates from TTVs imparted to the planet by the moon’s gravitational influence^7^. Thus, a basic criterion one might suggest is that TTVs should be present. However, for 25 of our targets only two transits were available and thus TTVs cannot be inferred. For the remainder, we apply a statistical test for TTVs as described later.

The other observational consequence of an exomoon is that its shadow can eclipse either the star or planet (that is, a syzygy), leading to additional changes in flux on top of the conventional transit signature^36^. These changes can occur in or out of transit and impart complex shapes. Furthermore, the limited number of transits available in our sample means that stacking approaches^82,83^ will not be statistically valid. However, our photodynamical planet–moon model (model ${{{\mathcal{M}}}}$) computed using LUNA does fully account for the moon’s subtle influence on the light curve. Since MultiNest provides marginal likelihoods, we can evaluate the Bayes factor between models ${{{\mathcal{P}}}}$ and ${{{\mathcal{M}}}}$, while correctly penalizing the moon model for its greater complexity, to determine the statistical evidence for a moon. We thus demand that the Bayes factor formally favours the planet–moon model over the planet model to be considered further.

Finally, we require that the planet has an orbit that is consistent with a circular path. Elliptical orbits can be produced through planet–planet scattering^84^, which is expected to strip exomoons^29^. Even if the eccentricity is produced through some other effect, the dynamical region of stability is severely truncated by non-zero eccentricity^28^. Thus, although an elliptical orbit does not prohibit exomoons, we consider it a priori improbable and thus reject any planets exhibiting eccentric orbits.

If these 2(+1) criteria are satisfied, the object is promoted for further checks, which we refer to as secondary tests (discussed in the next subsection). We describe the details of the initial tests in the following paragraphs and highlight that the results are listed in Supplementary Table 1.

For the eccentricity test, we require some formal criterion to evaluate if a planet has an eccentric orbit or not. Since we assume a circular orbit in all of our light-curve models, the derived mean stellar density will be offset from the true value if the orbit is in fact eccentric^85^. If we have an independent and unbiased measure of the mean stellar density in hand, this offset can be detected and thus used to constrain the orbital eccentricity^86^. Again, the philosophy here is to minimize the exomoon false-positive rate at the expense of the false-negative rate.

Our eccentricity test thus begins by taking the a posteriori parameter samples from either model ${{{\mathcal{P}}}}$ or ${{{\mathcal{T}}}}$—specifically, we default to ${{{\mathcal{P}}}}$ unless we conclude the system is ‘TTV hot’ as defined by our TTV test described later. Naturally, for two-transit planets we always use model ${{{\mathcal{P}}}}$.

Next, we need an independent measure of the true stellar density and here this originates from an isochrone analysis. This is described in a dedicated section later and results are summarized in Supplementary Table 2.

To determine an eccentricity posterior for each planet, we begin with the asterodensity profiling relationship^85^, which relates *ρ*_⋆,circ_ to the true value, *ρ*_⋆_:

1 $${\rho }_{\star ,{{{\rm{circ}}}}}\simeq {\rho }_{\star }{{\varPsi }},$$

where

2 $${{\varPsi }}\equiv \frac{{(1+e\sin \omega )}^{3}}{{(1-{e}^{2})}^{3/2}}.$$

Since *ρ*_⋆,circ_ and *ρ*_⋆_ are inferred independently, we construct a *Ψ* posterior by simply drawing random samples from our light-curve-derived density and dividing them by random samples from the isochrone density. This *Ψ* posterior sadly does not trivially lead to an eccentricity posterior because of the joint dependence on *ω*, the argument of periastron. Thus, we need to again sample the parameter space. To achieve this, we used kernel density estimation (KDE) on the log *Ψ* posterior with a Gaussian kernel and a bandwidth optimized for using least-squares cross-validation. The KDE distribution was then used as a log-likelihood function for the purposes of an MCMC exploration in {*e*, *ω*} parameter space.

Since transiting planets are more likely to be eccentric, a priori, as a result of geometric bias^87,88^, it is necessary to account for this selection bias during the inference. This in turn requires a prior for the eccentricity distribution, which cannot be strictly uniform to avoid infinities^89^. We thus assume that Pr(*e*), the prior on eccentricity, is a beta distribution with shape parameters *α* = 1 and *β* = 3, broadly matching the long-period radial velocity population^90^. The selection effect inherent to the transit method is then accounted for using the joint prior, Pr(*e*, *ω*∣transiting) as derived for eccentric planets^89^.

We then sampled the {*e*, *ω*} parameter volume 110,000 times, burning out the first 10,000 steps. Note that MCMC sampling suffers from biases at boundary conditions, such as *e* > 0, and this can lead to an artificial positive skew in eccentricity^91^. This can be overcome by reparameterizing^92^ to √*e* sin *ω* and √*e* cos *ω*, which we use here. Once the eccentricity posterior has been evaluated, we next perform Bayesian model selection by evaluating the Savage–Dickey ratio^93^. This simply evaluates the posterior density at *e* = 0 versus the prior, where the ratio provides a direct estimate of the Bayes factor of an eccentric versus circular orbit (in the case of nested models such as here). In our case, any instance where the posterior density is less than the prior at *e* = 0 is hereby labelled as ‘eccentric’, otherwise ‘circular’. The prior density is analytic and thus trivial to evaluate at zero^89^ but for the posterior density we apply a KDE to the posterior to evaluate the density at zero. Because of the boundary condition at *e* = 0, we mirror the posterior samples around zero and combine them with the originals, and then apply a Gaussian KDE to the combined sample. The density at zero is then equal to twice the density of this KDE at zero, as a result of the doubling of the sample volume.

Thus far, the eccentricity test described above has been applied to either the planet-only or planet-with-TTVs light-curve model, depending on whether we classified the planet as TTV hot (see next paragraphs). However, we also repeated this a second time applied to the planet–moon posteriors from model ${{{\mathcal{M}}}}$. If the planet appears incompatible with a circular orbit only after the moon component is introduced, we mark this with the ✘^†^ symbol in Supplementary Table 1 and the object is not considered further as a viable moon candidate. Such cases essentially mean that the required moon solution demands a light-curve shape that is inconsistent with the derived stellar density unless eccentricity or blending is introduced^94^.

We emphasize that transit planets preferring eccentric orbit solutions are identified via the existence of a ‘photoeccentric effect’, which describes an apparent tension between the light-curve-derived stellar density and an independently inferred value^86^. However, blends and starspots can also cause substantial tension, and both would lead to an elevated risk of exomoon false positives, motivating their exclusion. Finally, although we exclude these systems from this study, that does not mean they are necessarily devoid of moons, any more than hot Jupiters are necessarily devoid of moons. However, in both cases, physical arguments suggest that they are not the most suitable environment. Therefore, although we elect to avoid such systems in this study, efforts by other teams to survey such objects are by no means futile and we encourage such work.

For the TTVs, as noted earlier, 25 of our targets have only two transits available and thus cannot be tested. This is because TTVs are defined as an excursion from a linear ephemeris fit, but a linear ephemeris model (governed by two free parameters) will always provide a perfect fit to two arbitrary transit times (two data points). For the other cases, we can search for TTVs as an indication of an exomoon, as well as providing some novel insights about the propensity of cool giants to exhibit TTVs more broadly.

Testing for TTVs through periodogram searches is impractical for the vast majority of our sample. This is because 51/72 of our planets have three transits or fewer and thus will offer just three data points for a regression. For a sinusoidal TTV, the simplest periodic function, five unknown parameters describe the ephemeris (the period and reference time of transit minimum, as well as three sinusoid parameters—period, phase, amplitude). Even in a grid-search periodogram, which removes one parameter, TTV period, we still have fewer data than free parameters. Note that if we possess four data points the system becomes constrained but fits are typically ‘perfect’, although in such cases we can apply regularization techniques on the amplitude term to make progress, as has been done, for example, for Kepler-1625 b^95^.

Instead of trying to seek a periodic TTV, we simply ask whether there is evidence for a TTV. To this end, we follow earlier work^96^ and apply their first test that addressed this question. This takes the maximum-likelihood light-curve fits of models ${{{\mathcal{P}}}}$ and ${{{\mathcal{T}}}}$ and compares their log-likelihood through a BIC evaluation^68^. By working with the light curves directly, rather than derived products such as marginalized transit times, we are able to extract as much information from the light curve as possible. Any planet with a BIC preference for model ${{{\mathcal{T}}}}$ is labelled as TTV hot, else TTV cold, unless only two transits exist, in which case we use ‘TTV grey’.

### Secondary checks for exomoon candidacy

If a planet passes the basic checks described in the last section, we apply additional checks to evaluate the plausibility of an exomoon. In total, 11 of the 70 cool giants satisfy this criterion. First, we require that the planet’s eccentricity, as determined from model ${{{\mathcal{M}}}}$, also favours a circular path. Following the same method as described in the last section, we find that all 11 indeed appear consistent with circular after applying this test.

Next, we regressed a new moon model to the data, model ${{{\mathcal{X}}}}$, which is identical to model ${{{\mathcal{M}}}}$, except that negative- and zero-radius moons are permitted. Negative radii correspond to inverted transits and are simply implemented by flipping the signals. Zero-radius moons are formally forbidden in model ${{{\mathcal{M}}}}$ since we impose the density constraint that *ρ*_S_ < 20 g cm^−3^ and a zero-radius moon has infinite density. Thus, to enable this we relax this condition by simply commenting out this check in our code. Using the posteriors of model ${{{\mathcal{X}}}}$, we apply three statistical additional tests to the 11 objects.

The first of these, which could be labelled test 4 by this point, is that we computed a Savage–Dickey ratio at the location of *M*_S_/*M*_P_ = 0 to evaluate the statistical evidence in favour of a non-zero exomoon mass. If the system had three or more transits, we imposed the constraint that the Bayes factor from this calculation must exceed 10 in favour of a finite mass (that is, strong evidence^27^). Next, test 5, we computed a Savage–Dickey ratio at the location of *R*_S_/*R*_P_ = 0 and demanded that, for all objects, the Bayes factor preference for a non-zero radius exceeds 10. Finally, test 6, we counted up how many of the *R*_S_/*R*_P_ samples were negative versus positive and demanded that the positive:negative ratio exceeded 5. This final test catches the possibility that *R*_S_/*R*_P_ is offset from zero but has substantial weight in the unphysical negative-radius regime.

After applying these cuts, three objects emerged as possible candidates: KIC-8681125, KIC-7906827 and KIC-5351250 (aka Kepler-150).

### Vetting of KIC-8681125.01

Advanced vetting of the KIC-8681125.01 planetary moon candidate begins by visual inspection of the transit light-curve fits to better understand what type of moon signal is seemingly detected. As shown in Extended Data Fig. 2, the signal is remarkable for featuring no clear moon-like transit. Instead, the main difference observed is a transit depth change from $3{,}59{0}_{-130}^{+160}$ ppm to $3{,}03{0}_{-110}^{+140}$ ppm. This is explained by the moon model by placing the moon transit on top of the planetary transit in the first epoch, but then the moon avoids transiting the star altogether in the next epoch.

This situation was immediately suspicious and appeared somewhat convoluted and fine-tuned, particularly when we compare it with typical planet–moon models generated in simulation work^36^. One possibility is that a nearby contaminant source is more prominently included within the aperture of the second epoch than the first, thus diluting the second’s transit depth. However, if the source landed on the same silicon with the same aperture used each time, this would clearly be excluded as a possibility.

To investigate this, we used the Kepler target pixel files to inspect the photometry at the pixel level. Since the first epoch occurs in quarter 10, but the second in quarter 16, the spacecraft has rolled into a distinct position between the epochs (every four quarters it returns to the same position). As a result, the source is on different silicon between the two epochs. However, KIC-8681125 is located near the centre of the entire detector array, within module 13, and thus ends up remaining within this module even after the roll, since the roll is itself uses an axis with an origin close to the centre of the detector array. Despite this, it does indeed end up on different silicon moving, from quadrant 4 to quadrant 2 between the two epochs.

The optimal aperture used by the Kepler pipeline is also quite distinct between the epochs, as shown in Supplementary Fig. 1. Epoch 1 has a simple 2 × 2 square pixel centred on the source, but epoch 2 uses a ‘+’ shaped aperture with an extra pixel included in one corner. In total, six pixels are used in the second aperture, thus increasing the chance of a contaminant falling within the aperture. On this basis, we consider the hypothesis of a contaminant driving the depth change as being highly plausible.

To investigate further, we fitted the light curve with a model that was identical to the planet-only model except for the fact that the second epoch had a unique blend factor associated with it, *γ*. The maximum likelihood of this fit did not exceed the moon model, but it led to a major Δ*χ*^2^ = 50 improvement over the planet-only model. Since the model only requires one extra parameter over the planet-only model, whereas the planet–moon model needs seven, the blend model outperforms the moon model in terms of the marginal likelihood. As a result, it is formally the preferred model by a Bayes factor of 6.8.

While the blend hypothesis seems to naturally resolve this system, we highlight that problems still remain with this idea. Unfortunately, no high-resolution imaging has been previously obtained, but Gaia can resolve sources more than 1–2 arcsec away. The closest source (id 2127184090671914880) is 11.8 arcsec away and 1.7 mag fainter. Given the pixel scale of Kepler of 4.0 arcsec, this is probably too far away to explain the relatively large depth change, as well being somewhat fainter than expected to explain the depth change. Another possibility is that an unseen source resides closer, within approximately 1 arcsec of the source, evading Gaia. However, this is also not satisfactory, as the contaminant should then be sufficiently close as to be included in both epoch apertures. The contaminant hypothesis is thus challenged by the lack of an obvious known source.

We also considered the possibility that the star may be covered in spots, and between the two epochs the spot coverage varies to manifest the depth change. However, high spot coverage appears incompatible with the Kepler photometry, which is relatively flat. To explore this, we ran a Lomb–Scargle periodogram on each quarter and found that the amplitude is consistently below 200 ppm (Supplementary Fig. 2).

Other possibilities still remain, such as uncorrected stray-light video cross-talk, for example, but it will be difficult to make further progress in the absence of high-resolution imaging, which we encourage at this time. However, given our generally conservative approach of seeking reasons to reject moon candidates rather than keep them, sufficient reason for scepticism exists about this object that we do not consider it further as an exomoon candidate.

### Vetting of KIC-5351250.06

In vetting the planetary moon candidate of KIC-5351250.06/Kepler-150 f, we begin by noting that the star is unusually active amongst the sample considered. This is apparent from simple inspection of the light curves but also it has been previously reported^35^ as a rotationally active star with a periodicity of 17.6 d and an amplitude of 10.9 mmag (approximately 1%). Since the transit depth of Kepler-150 f is ~1.5 mmag, this implies that the area of the stellar surface covered by spots is larger than the sky-projected area of the planetary disk. Accordingly, it is quite possible for the planet to cross over one or more spots during the transit and induce upward flux undulations^34^ that mimic the signature of star–planet–moon syzygies^36^.

If the spots are much colder than the photosphere, then the spot crossings can be up to the entire transit depth. In practice, this is somewhat rare for even the most active stars^97^, requiring both a very cold spot and a perfect alignment of the spot and planetary transit chord^98^. On this basis, we proceeded with caution given the enhanced possibility of false positives.

To investigate further, we ran a Lomb–Scargle periodogram of the PDC Kepler data, quarter by quarter. As shown in Supplementary Fig. 3, we confirm the ~1% level activity reported previously^35^ and note that the activity seems greater in Q12 (corresponding to the second transit epoch of Kepler-150 f) versus Q5 (the first epoch). This indicates that spots are more likely to corrupt the second transit than the first.

Inspection of the transit light curve itself, shown in Extended Data Fig. 3, reveals an apparent transit depth change from epoch 1 to 2, going from $1{,}35{0}_{-190}^{+260}$ ppm to $1{,}10{0}_{-100}^{+90}$ ppm. Closer inspection reveals that the trough of the second transit is not uniformly higher, but rather bounces up and down sporadically—consistent with the behaviour expected for spot crossings^98^. Given that this transit coincides with an episode of high activity, this begins to cast doubt on the reality of the exomoon signal.

To go further, we fitted just the first epoch in isolation with a planet-only model and then used its maximum-likelihood solution as a template for adding starspots for the second transit. If the planet is passing over a spotty, dark patch—as we hypothesize—then the transit will also be diluted in depth because it is only now blocking out a relatively dim region of the star’s total intensity^98^. Thus, the second transit is modified in two ways: (1) the addition of a dilution factor, *γ*, and (2) the inclusion of *N* spot-crossing events. Since we are not particularly interested in the properties of the spots themselves, just whether they can fit the light curve better than a moon, we adopt a simple heuristic model for the crossings. Specifically, we add on a Gaussian of width *σ*, amplitude *A* and central time ${\mathbb{T}}$, thus meaning we have three parameters per spot.

In total, we regressed four different versions of this model to the second epoch: (1) no spot crossings but a contamination factor (one extra parameter), (2) one spot crossing and the contamination factor (four extra parameters), (3) two spot crossings and the contamination factor (seven extra parameters) and (4) three spot crossings and the contamination factor (ten extra parameters). Since the planet-only model has seven native parameters, the final model includes 17 variables altogether. The results of these fits are shown in Extended Data Fig. 3, along with the fits from the planet-only and planet–moon models.

For the planet-only and planet–moon models, we have been thus far comparing models using the Bayesian evidence. However, here, we seek an alternative model selection method. To see why, consider that in the cases of the planet–moon and planet-only models the model parameters are have physical meaning and thus have well defined parameter limits. For example, the moon’s orbital period is bounded by the inner Roche limit and the outer Hill sphere. In contrast, our heuristic model has no clear bounds on the parameters of interest. Thus, we could just increase the widths of the priors somewhat arbitrarily, which would then dilute the Bayesian evidences. Accordingly, the model selection results become highly subjective when using marginal likelihoods for heuristic models, and we instead prefer to use a model selection metric that compares the maximum-likelihood solutions, for which there is no sensitivity to prior widths.

The two most commonly used maximum-likelihood model comparison metrics are the BIC^68^ and the Akaike information criterion, or AIC^99^. The AIC—motivated from information theory—is more appropriate when none of the models are considered truly correct, but we are ranking them in terms of their ability to approximate the truth, which is certainly true for heuristic models. Further, the BIC includes a penalty term, which depends on the number of data points, and this introduces a degree of subjectivity into the model selection process since it depends on how much we window the data around each transit mid-point. For these reasons, we used the AIC to rank these different models.

In doing so, we found that the two-spot model is favoured with AIC improvements versus model (1) of 2.6, 8.0 and 4.9 for models (2) to (4) respectively. For the two-spot model, the *χ*^2^ score when computed on both epochs is 702.89, whereas the planet–moon model achieved 712.07. In other words, the two-spot model is a better match to the light curve than the planet–moon model by Δ*χ*^2^ = 9.2, despite using the same number of free parameters.

At this point, we could go further and introduce astrophysical spot models, coupled to the rotational modulations, but for the purposes of this work—seeking exomoons—this is simply beyond the scope of our objectives. Although we cannot fully reject the hypothesis of an exomoon, for the reasons described there is now sufficient basis to reject this particular candidate as a compelling object.

### Robustness of KIC-7906827.01’s moon signature against detrending choices

A possible concern with any claimed moon-like signal is that it is sensitive to the choices of detrending method used to process the data. In this work, we use the method-marginalized light curves, computed as described earlier, for the model comparison tests. Since this uses the median of eight different light-curve detrendings, it is possible that the signal is present in the majority of these, but not all. This does not necessarily indicate that the moon-like signal is spurious, but it would certainly motivate a deeper investigation as to why this is happening and increases the possibility of a spurious origin.

We therefore decided to inspect the individual detrendings for evidence that the signature of the exomoon candidate was not a global feature. This is complicated by the fact that the moon-like signal is not a single event, but rather presents itself in both transits through subtle distortions. Although a visual inspection of the light curve reveals broadly consistent morphologies across all methods (Extended Data Fig. 4), we sought a more quantitative metric to assess this.

To this end, we took the maximum a posteriori fit of the planet–moon model conditioned upon the method-marginalized light curve as a template, and compared it with each of the eight detrended light curves. For reference, we also took the maximum a posteriori planet-only fit. Crucially, we do not refit any of these eight light curves; we simply ask how well these templates agree with the data in hand. In every case, we find that the planet–moon model yields superior agreement, indicating that the specific signature of the hypothesized moon (and not some generic moon) is present in all detrendings. Furthermore, the Δ*χ*^2^ values obtained are consistent with the value obtained from the method-marginalized light curve (Δ*χ*^2^ = 23.2), yielding 22.2, 27.1, 23.4, 25.0, 23.9, 31.4, 23.8 and 15.0. This list has a median of 23.9, and a mean of 24.0 ± 4.6—consistent with the value obtained from the method-marginalized light curve.

On this basis, we conclude that the moon-like signature is robust against choice of detrending method.

### Pixel-level analysis of KIC-7906827.01

We analysed the pixel-level data of KIC-7906827 to look for anything out of the ordinary that might suggest that the moon-like signal is spurious. To this end, we largely follow the approach outlined in a previous work^37^, where a putative exomoon around Kepler-90 g was shown to be a likely false positive. This also builds upon the tests already shown for KIC-8681125.01 discussed previously.

We begin by extracting the individual raw light curves of each pixel within the postage stamp of the target and for times directly surrounding the two transits of KIC-7906827.01. Specifically, we extracted light curves of ±2.5 transit durations around the two known events. Each light curve was then detrended using the local method described earlier.

Next, we measure the planet SNR in each pixel by simply calculating the weighted mean of the detrended pixel light curves inside/outside the transit region (where we use the duration as determined from the full planet-only fits found earlier). The s.d. values (divided by the square root of the number of data points in each section) are used to compute an error (through quadrature), which then forms the SNR. The result is illustrated in the middle panel of Extended Data Fig. 5. In comparing with the mean flux counts of each pixel (shown in the left-hand panel of Extended Data Fig. 5), we see good agreement between the location of the highest flux and the location of the highest transit SNR. The planetary transit thus shows no sign of being dislocated from the target or any other strange pixel behaviour.

Turning now to the moon-like signal, we seek to replicate the SNR test, but this is challenged by the fact that the moon signature is not a simple box but rather displays features across the light curve, and in different positions in each transit. The SNR can instead be measured by asking, in each pixel, how much better the maximum a posteriori planet–moon light-curve model template is versus that of the planet-only model. Here, ‘template’ refers to the solution obtained by regressing to the method-marginalized light curves. To quantify what we mean by ‘better’, we evaluate the Δ*χ*^2^ between the two templates, such that positive numbers indicate that the planet–moon model leads to improved agreement.

As the moon-like signature inherently has much lower SNR than the planetary signal, the SNR map is correspondingly noisier, but it clearly shows a concentration of the SNR on top of the target, as expected for a genuine signal. We highlight that it is precisely this point that the moon candidate of Kepler-90 g failed to pass^37^. On this basis, we find no evidence in the pixel-level data to suspect that the moon-like signature (1) is associated with a contaminating offset source, (2) is caused by a global dimming of the detector postage stamp pixels (for example due to stray light) or (3) is caused by a single pixel triggering a false positive through anomalous behaviour.

### Centroid analysis of KIC-7906827.01

From the KIC-7906827 fits files, we extracted the flux-weighted centroid columns and inspected the time-series behaviour of the *X* and *Y* positions within the vicinity of the two transit epochs of KIC-7906827.01. Masking the transits themselves, and filtering on only data within six transit durations of the eclipses, we fitted a series of polynomials through the centroids of increasing complexity. Scoring with the BIC^68^, we identified the most favourable model for each transit in both *X* and *Y* and used this to remove the long-term trend caused by pointing drift.

We then evaluated the mean position in and out of the transit event, using the s.d. to estimate uncertainty, to find that the centroids exhibit a {−0.52 ± 0.06, +0.62 ± 0.05} millipixel shift in the {*X*, *Y*} directions (Supplementary Fig. 4). Given the presence of nearby stars observed by Gaia, a centroid shift of some kind is not surprising but it can also indicate that the transit is not associated with the target^39^—which would open the door to KIC-7906827.01 being a false-positive planet.

To investigate the possibility that one of the other known stars was in fact the host, we created and modelled difference images for the high-SNR transit events in quarters 8 and 16 for KIC-7906827. The results of this very strongly show that the observed transit signal cannot be due to any stars in the Gaia catalogue except the target star, KIC-7906827.

We created the difference image as described in previous work^39^. Assuming that all flux change is due to the transit event, the difference image will show a star-like image at the location of the transit signal source. For each quarter, cadences were chosen in the transit event and the pixel values were averaged over these cadences, creating an average in-transit image. The same number of cadences was chosen on both sides of the transit event, and averaged to create an average out-of-transit image. These observed images are compared in Supplementary Figs. 5 and 6. The similarity between the out-of-transit and difference images very strongly indicates that KIC-7906827 is the source of the transit.

Even greater confidence in this arrives via modelling of the point response function (PRF). We modelled the scene using the Kepler PRF and stars returned by a Gaia catalogue cone search with radius 12.8 arcsec as described in earlier work^100^. This search returned five stars, as dim as gmag = 21.0. The Gaia proper motion-corrected positions of these stars are plotted in all figures. The stars are placed at pixel locations using Kepler’s raDec2Pix code (https://github.com/stevepur/Kepler-RaDex2Pix).

Supplementary Figs. 7 and 8 compare the observed and modelled pixels, demonstrating the quality of the PRF modelling. Supplementary Figs. 9 and 10 compare the observed difference image (top left) with the modelled difference image assuming that the transit is on each of the five stars in the cone search. These simulated difference images were created by subtracting simulated scenes similar to Supplementary Figs. 7 and 8, with the in-transit scene reducing the flux of the modelled star by a fitted depth.

It is clear from Supplementary Figs. 9 and 10 that a transit on the target star is the only one that remotely matches the observed difference image. The other stars in the Gaia catalogue cannot reproduce the observed signal.

While this analysis excludes the possibility that a different known star hosts the transit signal, it does not address the possibility of an unknown star blended with the target. To investigate this, we first measured the position of the target star by performing a multistar PRF MCMC fit to the average out-of-transit image, and the position of the transit signal source by performing a single-star PRF MCMC fit of the difference image. These fits computed the posterior distribution of pixel position and flux for each star consistent with the data, and used a Gaussian likelihood for each pixel with width given by the propagated per-pixel uncertainty of the fitted image. These measurements are differenced to give the distance of the transit source from the target star. Measuring both the target star source and transit source with PRF fitting mitigates possible bias due to PRF error, because the same bias probably occurs for both stars.

The blend probability is computed using equation (14) of Morton’s earlier work^101^, which gives the probability of a blend that can mimic any planet within 2 arcsec of the target star as a function of the star’s Galactic latitude and Kepler magnitude (caution: the columns in Table 1 of ref. ^101^, which gives the coefficients for their equation (14), are reversed). For our star, the probability of a planet-mimicking blend within 2 arcsec is 3.08 × 10^−4^. We compute the 3*σ* radius of the target star on the basis of the 68th-percentile credible interval from the fit to the difference image, and scale the blend probability by the ratio of the 3*σ* circle to a 2 arcsec circle.

The results are summarized in Supplementary Table 3 for quarters 8 and 16. The transit depth is recovered by taking the ratio of the fitted fluxes of the difference image to the target star from the out-of-transit image, demonstrating the success of the fit. The transit source is about 70 mas from the target star, which is just over 1*σ*. The resulting blend probability is about 2.6 × 10^−6^.

### Statistical validation of Kepler-1708 b

Our centroid analysis establishes that the transit signal is associated with the target star and that a blend is highly improbable given current observations. This, in isolation, provides a compelling case that KIC-7906827.01 is a genuine planet. This possibility, often dubbed PRF contamination, dominates the catalogue of known Kepler false positives; for example, 1,587 of the 1,859 false positives identified through ephemeris matching^102^ to known eclipsing binaries originate from PRF contamination^56^. However, eclipsing binaries can also occasionally cause false positives without PRF contamination, via column anomalies, cross-talk and reflections^102^. However, we note that KIC-7906827.01 has already been tested for an eclipsing binary ephemeris match in the aforementioned work^56^ and no matches were found, further strengthening the case that KIC-7906827.01 is a genuine planet. To finalize this, we took the shape of the transit light curve, in combination with the stellar parameters, to independently validate KIC-7906827.01.

To this end, we used the vespa package developed for precisely this task^40^. Here, the shape of the transit light curve is compared with a suite of models including both planet and false-positive scenarios, to evaluate the statistical probability of each. The a priori probability of blending, on the basis of the star’s position and fundamental properties constrained from spectroscopy and Gaia, is used to weigh these scenarios appropriately in the final evaluation. One additional piece of information that can be helpful in this task is the existence and upper limit of an occultation event. A long-period planet like this should not produce a detectable occultation, and so its existence would put pressure on the planet hypothesis.

An occultation event is generally expected to be of approximately the same duration as the transit, so we can exploit this feature to provide a non-parametric means of detrending all of the Kepler quarters. Specifically, we use a median filter where the bandwidth is set to three times the transit duration of KIC-7906827.01, which essentially acts as a low-cut filter removing all variability on timescales greater than this threshold. The detrended light curve was then phase folded onto the ephemeris of the transiting planet modulo a half-period shift.

For a circular-orbit planet, the occultation should occur at a folded time of precisely zero. However, orbital eccentricity effects cause the occultation to shift away from zero. Since the eccentricity is unknown, especially if we remain agnostic about whether the transit signal is truly associated with the target star, then the shift is also unknown. Accordingly, we created a uniform grid of possible times across the entire orbit, spaced by one-tenth of the transit duration.

At each grid point, representing a possible time of occultation, we first evaluated the s.d. within an interval equal to the transit duration. This number was then divided by the square root of the number of data points minus one, and thus represents the achievable precision on an occultation event of similar duration to the transit as a function of orbital phase. Although this precision score exhibits fluctuations as a result of data gaps and sampling effects, we find that it centres around a value of 62 ppm. Repeating using the median deviation as a more robust variance estimator yields 59 ppm. If no occultation effect is detected then, we would estimate a 3*σ* limit of <180 ppm. For much shorter-duration occultation events, this would be overly optimistic though, since the smaller number of data points would inflate the uncertainty. While this essentially approaches infinity for infinitesimal-duration events, we adopt an upper limit of 10^1/2^ times shorter, which corresponds to <330 ppm.

The above explicitly assumes no occultation event, which we have to demonstrate. To this end, we took each grid point and evaluated the SNR of an occultation at 20 different trial durations (0.1 to 2.0 times the transit duration in 0.1 steps). From these, we select the highest-SNR duration as the saved solution and continue to move through the grid of possible occultation times. In this way, a genuine detection would manifest as a high-SNR bump within the grid, where we define the SNR as the mean out-of-occultation minus the in-occultation intensity divided by the uncertainty on that mean (as computed using the s.d.).

For Kepler photometry, eclipses generally need to have SNR > 7 to be considered compelling^38^, and we find no values near this level. The highest recorded SNR amongst 9,225 realized positions with more than two data points within the interval was 3.0. We thus find no evidence for an occultation event of KIC-7906827.01. If KIC-7906827.01 were a real planet, this would be the expected result, since its long-period nature means it would be far too dim to be detected photometrically. From the grid, we can also estimate an upper limit on the occultation depth in an alternative way. Specifically, we evaluated max(*δ*_occ_, 0) + 3*σ*_occ_ at each grid point, where *δ*_occ_ is the occultation depth and *σ*_occ_ is the uncertainty. We then evaluated the median of this array and added three times the s.d. of the array. This is technically overkill as a 3*σ* limit, since we have used a 3*σ* limit twice, but nevertheless it yields <350 ppm as an upper limit. This is in good agreement with our <330 ppm value from earlier and thus we adopt 350 ppm as a 3*σ* upper limit in what follows.

Using this constraint with the light curve, stellar atmosphere properties and Gaia parallax, we used vespa to calculate the statistical probability of a false-positive scenario. Eclipsing binary, hierarchical eclipsing binary and blended eclipsing binary scenarios are all highly disfavoured and lead to a planet FPP of 1 in 4,237, or 0.024% (Supplementary Fig. 11). Combining this with the similar independent conclusion from the centroid analysis, we conclude that KIC-7906827.01 is a genuine planet to high confidence and thus refer to it as Kepler-1708 b in what follows.

### Exploring the possibility of alternative astrophysical models for Kepler-1708 b-i

The case for an exomoon rests upon the light-curve analysis of the Kepler photometry. In particular, the Bayes factor of 11.9 for the planet–moon model versus the planet-only model drives the exomoon candidacy, as it surpasses the strong-evidence threshold of >10 adopted in this work and recommended by previous works^27^. Bayes factors are influenced by the likelihood function and the priors. In this case, the priors do not have arbitrary bounds but rather well motivated physical limits (for example the longitude of the ascending node lives on a circle from 0 to 2π rad). Further, the case for an exomoon signal remains compelling when viewed in a purely likelihood-based framework, with a Δ*χ*^2^ = 23.2 improved fit, indicating a 4.8*σ* effect. On this basis, we argue that the likelihood function drives this result and is the place where we might rightfully apply sceptical interrogation.

The likelihood function can be wrong in two circumstances: (1) the forward model is wrong; (2) the noise model is wrong. We consider each of these in turn but in this section address the former.

Regarding the forward modelling, the models in question are those of a planet transiting a limb-darkened star versus a planet–moon transiting a limb-darkened star. We might well wonder if some other model is ignored that is truly responsible. In general, the asymmetric and short-term time-variable nature of the transit shape is difficult to explain with some other localized astrophysical effect associated with the planet. For example, a ring system^103^ would need high obliquity, precession and many times greater physical extent than Saturn’s rings to explain the data. Further, such an extensive ring system would compellingly distort the light-curve-derived mean stellar density from the true value in a manner not observed here^104^.

We performed an additional check to see if the timing of the two inferred moments of exomoon transit were suspicious or improbable. Exomoon transits should be located close to the planetary event, moving back and forth ostensibly randomly with a range governed by their semimajor axis around the planet. The probability distribution of times is expected to follow an arcsine distribution^83^. Although we only have two such times available, it is possible to evaluate a *P* value (‘surprisingness’ score), which might indicate tension with our choice of model (that is, the planet–moon model). To investigate this, we took our maximum a posteriori planet–moon and regenerated the light curve but randomized the phase of the exomoon. Repeating 1,000 times, we were able to determine that the moon transits could have occurred up to ±0.25 d either side of the transit, with a spread broadly following the arcsine distribution as expected. This may be compared with the observed times of exomoon transit minima, of −0.226 d and +0.136 d. Adopting the arcsine distribution, we can evaluate the formal likelihood of obtaining the two observed times, which was ${{\mathrm{log}}}\,{{{\mathcal{L}}}}=1.82$. To put this number in some context, we repeated the above but drew two random times from the arcsine distribution, evaluated their likelihood and built up a distribution of likelihoods under the null hypothesis. The distribution is shown in Supplementary Fig. 12, where one can see that the real likelihood score sits very close to the centre of the expected distribution and is thus not remotely surprising. Accordingly, the timing of the observed moon transits does not appear suspicious or offer grounds to reject the planet–moon hypothesis.

Aside from a localized effect, the light-curve model could be wrong if some other non-localized phenomena simply coincidentally occurred during the time of transit of Kepler-1708 b. The most obvious example would be a second transiting planet in the system. Given the local window used of ±6.2 d, the probability of this occurring is min(12.4/*P*_c_, 1) (depicted in Supplementary Fig. 13 by the green dashed line) and thus improbable for *P*_c_ ≫ 12.4 d. We note that there are no other known planetary candidates or even threshold-crossing events^56^ reported for this source. Nevertheless, this remains a possibility if the hypothetical planet were simply too small to have been reliably detected by the Kepler pipeline. Given the depth of the observed deviations, the planet would need to be 2.6 *R*_⊕_ in radius at some unknown period—so how possible is it that such a planet is hiding in the existing Kepler data?

To explore this possibility, we first regressed a two-planet transit model to the same data as used for the planet-only and planet–moon fits. Note that these data only locally detrend the time series to within ±6.2 d of the transit events—which we dub ${{{{\mathcal{D}}}}}_{{{{\rm{local}}}}}$ in what follows. We let the second planet have an unknown period with a log-uniform prior from 10 to 1,000 d and uniform priors for impact parameters, transit time within the first epoch’s window, and ratio of radii. Using MultiNest to explore the parameter space, the best-fitting solution yielded a *χ*^2^ substantially lower than that of the planet–moon model, by Δ*χ*^2^ = −16.2. Further, the two-planet model is only modestly improved over the one-planet model despite being a nested model with four additional free parameters (thus demanding an improved *χ*^2^) with Δ*χ*^2^ = +7.0. Indeed, this leads to the model having a worse marginal likelihood than the planet-only model, with ${{\mathrm{log}}}\,{{{{\mathcal{Z}}}}}_{{{{\rm{2-planet}}}}}-{{\mathrm{log}}}\,{{{{\mathcal{Z}}}}}_{{{{\rm{2-planet}}}}}=-0.94$. We note that this is well approximated by evaluating the AIC between the two models using the *χ*^2^ difference (yielding −1.05). Thus, we find that the local transit photometry, ${{{{\mathcal{D}}}}}_{{{{\rm{local}}}}}$, does not support the two-planet hypothesis.

We find that the posterior distribution for *P*_c_ almost replicates the prior of a log-uniform form. Thus, in any given log *P* window, we have approximately the same number of posterior samples. Exploiting this, we group the posterior into eight evenly spaced bins in log *P* space, with approximately 4,000 samples in each window. From these, we evaluate the maximum-likelihood solution amongst the subset. Since the AIC well approximates the marginal likelihood here, we use it to evaluate the Bayes factor as a function of log *P* at these eight grid points, which we then spline-interpolate to create a continuous function. The result is shown in Supplementary Fig. 13 (red dotted line), where we can see that long-period solutions are in greatest tension with the ${{{{\mathcal{D}}}}}_{{{{\rm{local}}}}}$ data.

The above only uses the photometry local to the Kepler-1708 b transits, but the broader complete Kepler time series would also be expected to exhibit transit signatures if the signal were caused by an interior transiting planet.

To support planet occurrence estimates from the DR25 Kepler planet-candidate catalogue^56^, the sensitivity for detecting a planet of a given period and radius was previously measured in detail^105,106^. The planet detection sensitivity was measured through Monte Carlo transit signal injection and recovery experiments^107,108^. In previous work^105^, a model was generated for planet detection sensitivity that depends on the stellar properties and noise characteristics of the Kepler flux time series based on fits to the database of transit signal injections. The planet detection sensitivity model can be calculated for any given Kepler target from the data products hosted by the NEA (https://exoplanetarchive.ipac.caltech.edu/docs/Kepler_completeness_reliability.html) and the accompanying KeplerPORTs Python software package (https://github.com/nasa/KeplerPORTs). Example uses of KeplerPORTs, in the context measuring planet occurrence rates, have been previously published^109–111^.

To calculate a planet detection contour for Kepler-1708, we use stellar parameters as updated in this study given in Supplementary Table 2. We adopt stellar limb-darkening parameters for Kepler-1708 (0.428, 0.4356, −0.1019, −0.0394; four-parameter nonlinear limb-darkening parameterization) by adopting the limb-darkening values from the target with the closest match in stellar properties in the DR25 Kepler stellar catalogue^54^. The photometric noise properties for Kepler-1708 are provided as supplemental columns in the DR25 stellar catalogue. Kepler-1708 was observed for 1,459 d with a duty cycle of 87%. The values of CDPP (combined differential photometric precision^112^) slope at short and long durations (−0.70477, −0.3524, respectively^105^) indicate a well behaved flux time-series data series for Kepler-1708 with minimal amounts of non-Gaussian noise. The window function and 1*σ* depth function data^113^ for Kepler-1708 were downloaded from the NEA.

The resulting planet detection contour from KeplerPORTs for Kepler-1708 is shown in Supplementary Fig. 14. If an additional planet had the proper inclination to transit, the detection contour provides the probability that a particular signal of a given period and radius would have been classified as a planet candidate in the final DR25 Kepler planet-candidate catalogue. As expected, the detection contour degrades toward small planets, as they have smaller transit depths, and at long orbital periods, as fewer transits are available to stack and enhance the detection SNR. The standard version of KeplerPORTs publicly available represents the recoverability of planet signals due to the Kepler pipeline by itself. An additional reduced sensitivity to planet signals can result from the vetting procedure^56,106,114^. The results shown in Supplementary Fig. 14 take into account the additional loss of sensitivity due to the vetting procedure. The vetting degradation was measured following a procedure similar to the description in section 4.2 of a previous analysis^111^.

While Supplementary Fig. 14 depicts the entire range of possible radii, for this study we are most interested in the radius slice corresponding to that necessary to explain the exomoon-like signal, namely 2.6 *R*_⊕_. Supplementary Fig. 13 (blue solid line) shows a slice in the detection contour plane at this radius as a function of orbital period. The discrete changes in detection probability (at 10, 60, 100, 200, 400 and 700 d) result because the pipeline detection probability and vetting recoverability probability are fitted independently over orbital period regions. The detection probability model fits are not required to be continuous across orbital period region boundaries.

The results described thus far can be combined to evaluate the overall probability that the observed moon-like deviations were caused by a second, previously undetected transiting planet. This is evaluated by taking the product of the three probabilities described thus far: (1) the probability that the second planet coincidentally transits during the local window used to regress Kepler-1708 b, (2) the probability that the two-planet model better explains the local data versus the one-planet model (in a Bayesian sense) and (3) the probability that a second planet of the required radius evaded detection from the Kepler pipeline. This combined probability as a function of orbital period is shown in solid black in the right-hand panel of Supplementary Fig. 13. As shown, the probability does not exceed 1% in the 10–1,000 d region considered. This result is somewhat overoptimistic in that does assign a prior probability for a such a planet existing in the first place and thus tacitly it is unity in the above calculation. On this basis, we find that the probability of a second transiting planet explaining the observed effects is ≲1%.

### Noise properties of the detrended Kepler-1708 b photometry

As discussed in the previous subsection, the likelihood function could be wrong if the forward model is wrong or the noise model is wrong. With the former investigated, we now turn to the latter. The data used to infer the exomoon candidate span ±6.2 d of the two transit events. These data were already prewhitened by virtue of the detrending process described earlier—specifically method-marginalized detrending. Since our likelihood function assumes independent Gaussian noise, the likelihood function adopted (and thus inferences thereafter) would be technically wrong if the noise were not described by independent Gaussian noise.

In reality, no observations are ever purely Gaussian. The removal of instrumental and astrophysical trends cannot ever be a perfect process. However, we hope to reach a state where the non-Gaussian component is much smaller than the Gaussian noise, and thus the likelihood function adopted can be treated as an excellent approximation. In this subsection, we thus investigate to what extent this appears to be true.

Using the method-marginalized time series we excluded the data within ±0.55 d of the times of transit minima, to trim the region where the planet–moon transits occur. The remaining data should now be described by a normal distribution. One of the simplest tests of this is to plot a histogram of the normalized intensities (upper panel of Supplementary Fig. 15). On this same figure, we plot the probability density function of a normal distribution centred on unity with an s.d. governed by the measurement uncertainties (that is, this is not a fit). As expected for Gaussian noise, we find excellent agreement. Further, a Kolmogorov–Smirnov test of the Gaussianity reports a *P* value of 0.10—consistent with expectation. Finally, the *χ*^2^ of these data against a flat-line model is equal to 1,073.8, in close agreement with the expected value given by the number of data points, 1,074. On this basis, the data do indeed appear normal.

Time-correlated noise can be difficult to identify using the tests described thus far and a better evaluation comes from looking at the temporal properties of the noise. Because of the jumps between each data segment, we split the data into four sections; epoch 1 pretransit, epoch 1 post-transit, epoch 2 pretransit and epoch 2 post-transit. The pretransit moon feature in epoch 1 corresponds to approximately eight cadences and thus we first tried a simple autocorrelation test at lag-8 on these four sections, which finds no compelling autocorrelation (*P* values of 0.54, 0.11, 0.73 and 0.28). We next tried a classic root mean squared binning test, where we bin the data sections into progressively larger bins and evaluate how the scatter evolves. Supplementary Fig. 15 (lower panel) shows the results compared with the expected behaviour of independent Gaussian noise, where again we find no clear evidence for time correlation.

On this basis, we conclude that the detrended time series appears consistent with independent Gaussian noise and thus the adopted likelihood function is appropriate.

### FPP of the exomoon signal Kepler-1708 b-i

An advantage of seeking exomoons is that the null hypothesis is well defined and can be injected into real photometric time series. Briefly, we can take the best-fitting planet-only model parameters, generate a template model and inject this into the SAP or PDC photometry as desired, and repeat the same detection process as outlined in this work. This allows us to directly calculate the FPP of detecting an exomoon-like signal.

We emphasize that this is not the same FPP as used earlier when validating Kepler-1708 b. There, the reality of the transit signal was unambiguous, but the underlying cause was uncertain. Here, the situation is somewhat reversed. The reality of the exomoon-like signal is unclear—and the FPP in this section seeks to address this. However, the underlying cause of the signal (assuming it to be true) is not addressed by such a calculation. This has already been addressed earlier, where we concluded that a second unseen transiting planet was the most likely astrophysical false positive, but even this has a probability of ≲1% of explaining the observations. In what follows, we focus on the FPP of the signal itself being astrophysical. Although we refer to this as ‘astrophysical’ hereafter, this is technically somewhat of a misnomer since we are really addressing the probability of time-correlated noise causing a false positive, which could in fact be considered astrophysical if due to stellar activity.

To inject fake planet signals, we took the maximum a posteriori parameters from the planet-only model fits of Kepler-1708 b to define a null-hypothesis template. Quarters 1 to 17 long-cadence data are available for KIC-7906827 as possible times into which to inject the signal. In some of these quarters, we observed discontinuities in the SAP time series (for example, due to pointing tweaks) and we went through and located these, saving to a library file. We then injected two transits of Kepler-1708 b, using the template with the only difference being that *τ* is randomized. The injection is performed by simply multiplying the SAP and PDC flux values by the template model (=1 outside transits and <1 inside). In this way, the time-correlated noise structure of the data is preserved.

There are several cases where the injections were rejected and reattempted. For example, if one of the transits is injected into a data gap (for example between quarters) the realization was rejected and retried. Our specific criteria for a ‘good’ injection were the following.

1. The injected transits must occur more than three transit durations away from the real transit (to avoid signal overlap).
2. The injection has to occur at least half a transit duration after the beginning of a quarter’s start time, and at least half a duration before the end of a quarter’s start time.
3. The trimmed (see below) transit epoch files have to contain at least 530 data points in each (ensuring injections have ≳90% of the data volume of the real signals).

The real transits were removed from the photometry, excluding data within two transit durations of the best-fitting transit times. The real analysis trimmed the photometry to within six transit durations of the central times, and thus we use the same trimming here. As with the original analysis, if a discontinuity occurs somewhere within the time series of interest, we only consider the continuous segments surrounding the transit in question. Finally, the number of 530 points was chosen since the original data have 591 (Q8) and 586 (Q16) points. We wish to ensure that the injections contain at least 90% of the smallest of these two (586), which in principle is 527 data points. However, we found that out outlier rejection algorithm rejects approximately 2% of the time series, and by this expectation we need 530 points to ensure the 90% threshold.

In total, we created 200 random injections (and thus 400 injected transits). We next applied the same method-marginalized detrending algorithm to all 200, with the only difference being that the GP method was dropped for computational expediency. The method-marginalization algorithm performs a final check for the Durbin–Watson statistic and root mean squared versus bin size behaviour, evaluating a *P* value against bootstrapped experiments. In some rare cases, this led to an epoch being rejected if none of the methods were able to produce sufficiently whitened time series. If this occurred, and fewer than two detrended transits were outputted, the realization was rejected and restarted with a new random seed.

The detrended light curves were then fitted using MultiNest coupled to LUNA, as before, trying both the planet-only and planet–moon models with identical priors (except that *τ* is shifted onto the new ephemeris). If the Bayes factor between the two models exceeded 10, denoting strong evidence, it was flagged as a possible candidate as with the real analysis, as shown in Fig. 3. For such cases, of which we found just three instances, the next step was to perform the negative-moon-radius test. Two of the three aforementioned cases (injections 103 and 161) pass this test, and we consider these to be ‘false positives’ from the suite of 200 injections. Their signal shapes are shown in Supplementary Fig. 16.

By requiring these signals to be viable moon candidates, in other words signals that our planet–moon model can explain as being physically sound, not all dips and bumps in the light curve trigger a false positive, only the plausible ones. For example, Fig. 2 shows a small deviation around BJD 2456341 that was not interpretable by our fits as a moon signal and thus does not constitute a false positive by this definition.

On this basis, we conclude that the false-positive rate of the exomoon-like signal of Kepler-1708 b is $1.{0}_{-1.0}^{+0.7}$% (uncertainty from counting statistics).

### Interpreting the FPP

Given that we looked at 70 exoplanetary candidates in this survey, one success from 70 with a 1% false-positive rate might at first seem to fully explain this event without invoking an exomoon. Certainly, this is a valid concern, and one we share. However, this tacitly assumes that the 1% false-positive rate computed for Kepler-1708 b holds for all of the exoplanetary candidates surveyed, which neither has been demonstrated nor can be reasonably assumed. Each case will have its own bespoke FPP dependent upon its specific time-correlated noise properties. Further, 45 of the 70 surveyed objects have three or more transits (unlike Kepler-1708 b) and thus have to also pass a TTV test, which would lower their false-positive rates by virtue of the extra check.

However, let us assume that the FPP (as caused by time-correlated noise) was indeed 1%, for the sake of making progress. In this case, it is important to stress that while one false positive is not an unexpected outcome, nor is zero false positives. Indeed, the likelihood ratio of the binomial distribution for *n* = 70 samples and *P* = 0.01 between *X* = 1 false positives and *X* = 0 false positives is 0.707. In other words, it is marginally more likely that a survey of 70 objects would produce no false positives than one. However, even this does not address what we really care about, which is the probability that this is an exomoon given the signal. Let us step back from the ensemble and look at Kepler-1708 b in isolation once again.

Let *E* stand for ‘has a Kepler-1708 b-i-like exomoon’ (and $\bar{E}$ means it does not), and *Q* denotes ‘passes our battery of tests’ (and $\bar{Q}$ does not). With this notation, we can express the probability we seek as

3 $$\Pr (E| Q)=\frac{\Pr (Q| E)\Pr (E)}{\Pr (Q)}$$

where Pr(*E*) is the a priori probability of an exoplanetary candidate in our sample having a Kepler-1708 b-i-like exomoon (that is, the underlying occurrence rate of such moons in the target sample). The denominator can be expanded as

4 $$\Pr (Q)=\underbrace{\Pr (Q| E)}_{\begin{array}{c} = {{{\rm{TPP}}}}\end{array}}\Pr (E)+\underbrace{\Pr (Q| \bar{E})}_{\begin{array}{c} = {{{\rm{FPP}}}}\end{array}}\Pr (\bar{E}),$$

where we have denoted the FPP and true-positive probability (TPP) explicitly. With some rearranging, we can show

5 $$\frac{\Pr (E| Q)}{\Pr (\bar{E}| Q)}=\frac{{{{\rm{TPP}}}}\,\Pr (E)}{{{{\rm{FPP}}}}(1-\Pr (E))}.$$

The TPP is essentially the completeness, and an accurate assessment is challenged by defining what we even mean by a ‘Kepler-1708 b-i-like exomoon’. However, given that the signal had a 4.8*σ* significance, we should generally expect a high TPP for such signals (TPP ≃ 1). Put another way, it would be odd if we missed these. A detailed calculation of the TPP is beyond the scope of this work and we argue largely unnecessary for the reasons stated above, as well as the fact that Pr(*E*) dominates our uncertainty in the calculations. If we set TPP → 1, then we have

6 $$\begin{array}{ll}\mathop{\lim }\limits_{{{{\rm{TPP}}}}\to 1}\frac{\Pr (E| Q)}{\Pr (\bar{E}| Q)}&=\frac{1}{{{{\rm{FPP}}}}}\left(\frac{\Pr (E)}{1-\Pr (E)}\right)\\ \mathop{\lim }\limits_{\Pr (E)\ll 1}\mathop{\lim }\limits_{{{{\rm{TPP}}}}\to 1}\frac{\Pr (E| Q)}{\Pr (\bar{E}| Q)}&=\frac{\Pr (E)}{{{{\rm{FPP}}}}}.\end{array}$$

From this, we estimate that $\Pr (E| Q) > \Pr (\bar{E}| Q)$ if Pr(*E*) > FPP. Here, then, if 1% or more of our sample host Kepler-1708 b-i-like exomoons, we should expect that the detected signal is most probably a real exomoon rather than a false positive. This calculation reveals the catch-22 conundrum facing the interpretation of this detection. In isolation, it is not possible to reliably assess the odds that it is real since we do not know the underlying occurrence rate of similarly sized moons around cold Jupiters.

Zooming back out to the ensemble, the total number of detections of Kepler-1708 b-i-like exomoons should be

7 $$\mathop{\sum }\limits_{i=1}^{70}{{{{\rm{TPP}}}}}_{i}\Pr (E)+{{{{\rm{FPP}}}}}_{i}.$$

In principle, we could define a likelihood function from this to infer Pr(*E*) based on our one success and see if it is consistent with zero—which would favour Kepler-1708 b-i being a false positive. However, assuming TPP_*i*_ ≃ 1 for all 70 is not well motivated here due to the different noise properties of each source, and similarly the FPPs will be distinct, as already discussed.

As this section establishes then, an accurate calculation of the probability of Kepler-1708 b-i being genuine is marred with challenges, stemming from the unknown occurrence rate of exomoons and the individual target FPP/TPP properties. This also extends to considerations of specific parameters of our retrieved fit versus false-positive scenarios. In principle, the FPPs and TPPs could be determined with far more extensive computational runs than done here, although we highlight that this study has already taken several years to complete and leveraged supercomputing time throughout (although not continuously). The enormous computational challenge, human time and CO_2_ production associated with such an endeavour has to be weighed against the benefits, or the simple act of just reobserving Kepler-1708 b in the future to more straightforwardly (and less ambiguously) address this question.

In conclusion, in considering the exomoon-like signal associated with Kepler-1708 b, we can find no firm grounds to reject it as a candidate at this time. Future supporting evidence could be found by detecting TTVs, predicted in the main text to have an amplitude between 1.2 and 77.0 min (95% confidence). In isolation, this would not be sufficient to confirm the moon due to the possibility of perturbing planets. In practice, we argue that the only real way to confirm/deny the existence of the moon convincingly would be high-precision transits of several future epochs, with the next event due on 24 March 2023 (BJD 2460027.86).

## Supplementary information

Supplementary Information Supplementary Figs. 1–16 and Table 3.

Supplementary Table 1 Initial (columns 2–4) and secondary (columns 5–7) exomoon candidacy tests applied to the 70 cool giants (column 1) in our survey. For each, we simply mark whether the test was passed/failed with a tick or cross. The dagger symbol denotes that the circularity test was only failed for the planet–moon model.

Supplementary Table 2 Fundamental stellar parameters inferred for the cool-giant host stars in our sample using an isochrone analysis. Values quoted define the median and surrounding 68.3% confidence interval of the posterior distributions.

## Data Availability

The data that support the plots within this paper and other findings of this study are made available at 10.5061/dryad.18931zcz9; or from the corresponding author upon reasonable request. Source data are provided with this paper.

## Source data

Source Data Fig. 1 Individual eccentricity constraints.
Source Data Fig. 2 Detrended photometry of KIC-7906827.
Source Data Fig. 3 Injected planet-only realization results.
Source Data Extended Data Fig. 1 Population eccentricity constraints.
Source Data Extended Data Fig. 2 Detrended photometry of KIC-8681125.
Source Data Extended Data Fig. 3 Detrended photometry of KIC-5351250.
Source Data Extended Data Fig. 4 Multimethod detrended photometry of KIC-7906827.
Source Data Extended Data Fig. 5 Pixel-level data for KIC-7906827.

## Code availability

The MultiNest regression algorithm^80^ is publicly available at https://github.com/farhanferoz/MultiNest. The vespa software package^40,101^ is publicly available at https://github.com/timothydmorton/VESPA. The isochrones software package^72^ is publicly available at https://github.com/timothydmorton/isochrones. The KeplerPORTs software package^105,107,113^ is publicly available at https://github.com/nasa/KeplerPORTs. The raDec2Pix software package is publicly available at https://github.com/stevepur/Kepler-RaDex2Pix.
