# Supplementary material for: An exomoon survey of 70 cool giant exoplanets and the new candidate Kepler-1708 b-i
Source: Nat Astron. 2022 Jan 13;6(3):367–80. doi: 10.1038/s41550-021-01539-1 (PMC8938273; doi:10.1038/s41550-021-01539-1)
Supplement: Supplementary file 1 — Supplementary Figs. 1–16 and Table 3. [file 41550_2021_1539_MOESM1_ESM.pdf]

nature portfolio

https://doi.org/10.1038/s41550-021-01539-1

Supplementary information

An exomoon survey of 70 cool giant exoplanets and the new candidate Kepler-1708 b-i

In the format provided by the authors and unedited

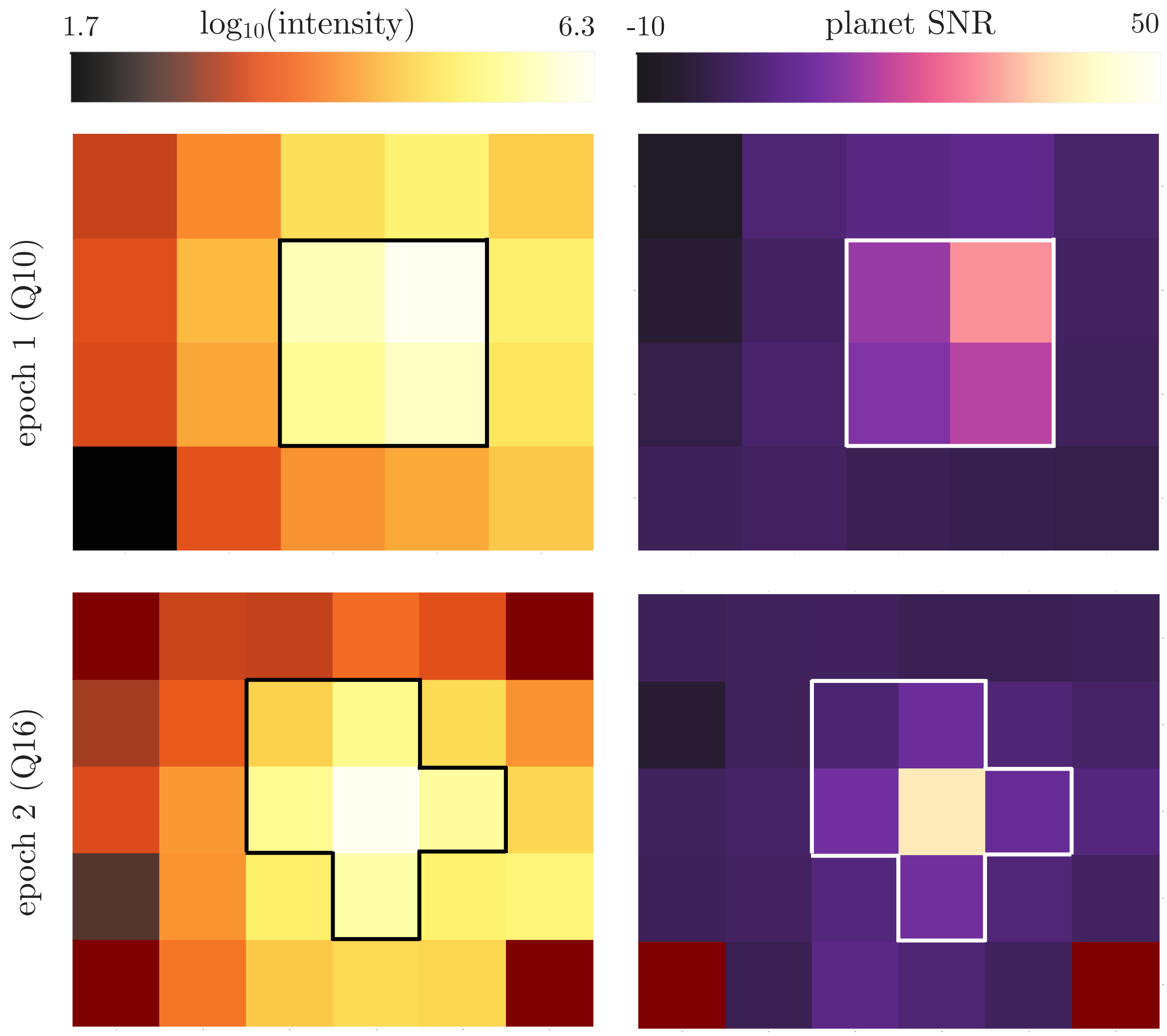

Supplementary Figure 1 | Comparison of the aperture used between the two transit epochs of KIC-8681125.01. Left: Pixel log-intensity is shown for the postage stamp downloaded for KIC-8681125 from the *Kepler* spacecraft, for epochs 1 (top) and 2 (bottom). The black solid outline shows the optimal aperture selected by the *Kepler* pipeline. Right: Same as the left, except we show the signal to noise ratio (SNR) of the transit signal in each pixel. As expected, the transit signal is co-located with the brightest source in view.

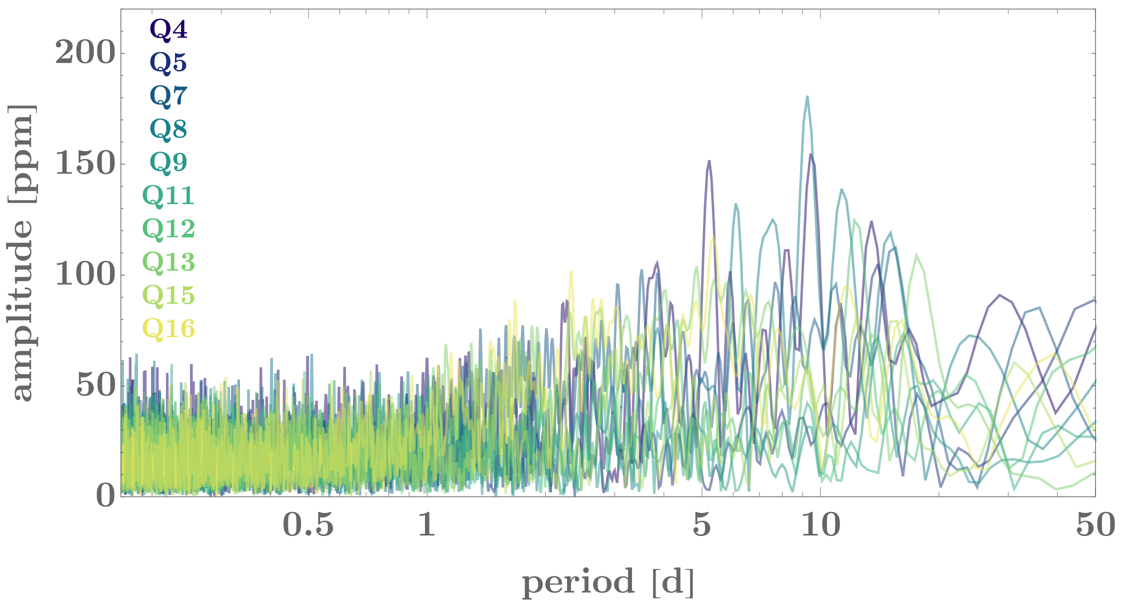

Supplementary Figure 2 | Lomb-Scargle periodogram of each available *Kepler* quarter for KIC-8681125. Colours delineate each quarter, as denoted by the legend. The amplitude appears bound to be less than 200 ppm for all quarters and thus relatively quiet.

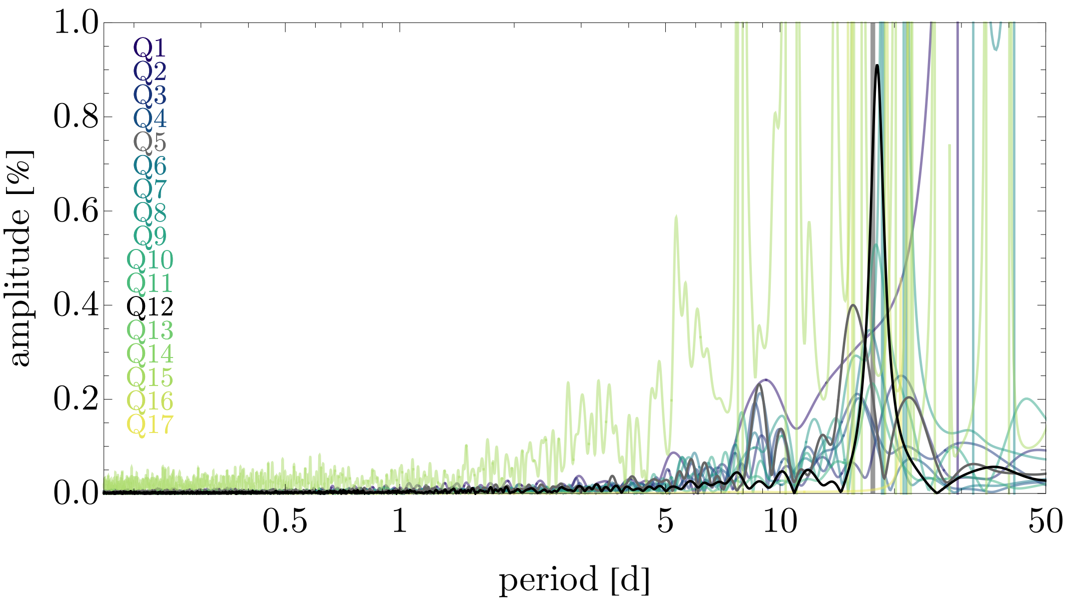

Supplementary Figure 3 | Lomb-Scargle periodogram of each available *Kepler* quarter for KIC-5351250/Kepler-150. Colours delineate each quarter, as denoted by the legend. The amplitude reaches up to 1%, and is particularly active in Q12 (highlighted in black) - corresponding to second epoch of Kepler-150f indicating that spots are more likely to observed then.

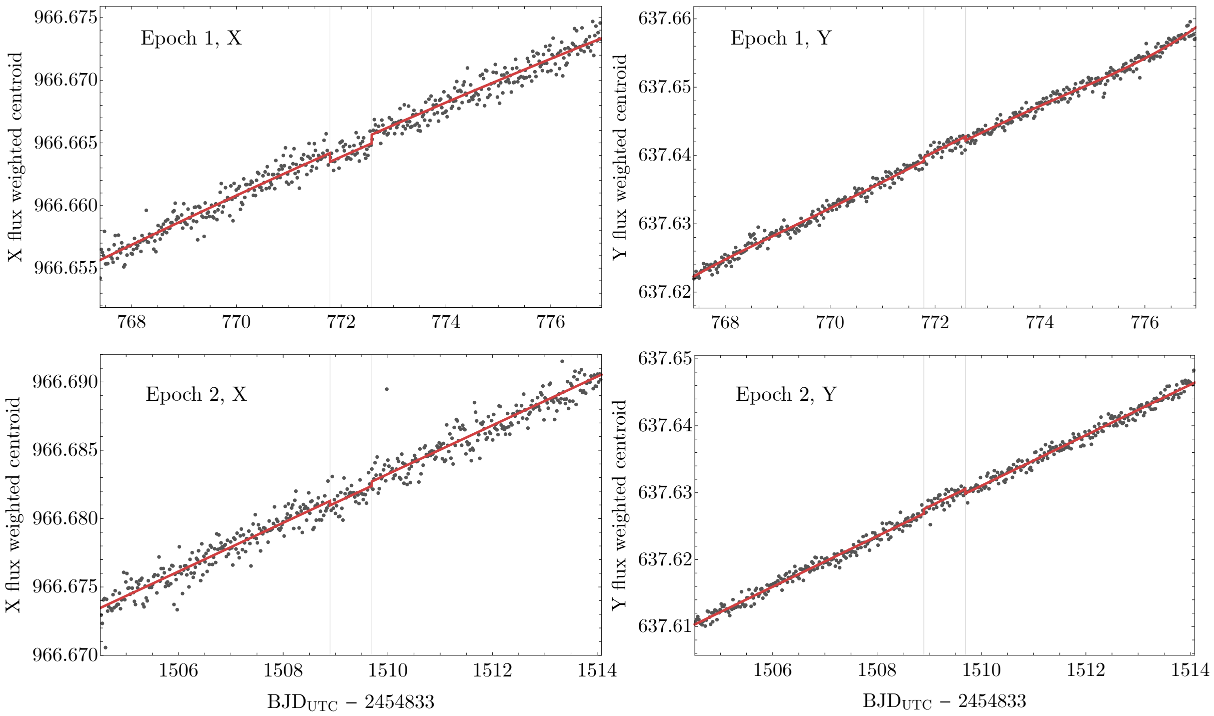

Supplementary Figure 4 | Flux weighted centroid time series of KIC-7906827.01. As visible from the plots, which are labeled in the top-left corner of each panel, the centroids exhibit a small shift during the time of the transits of KIC-7906827.01.

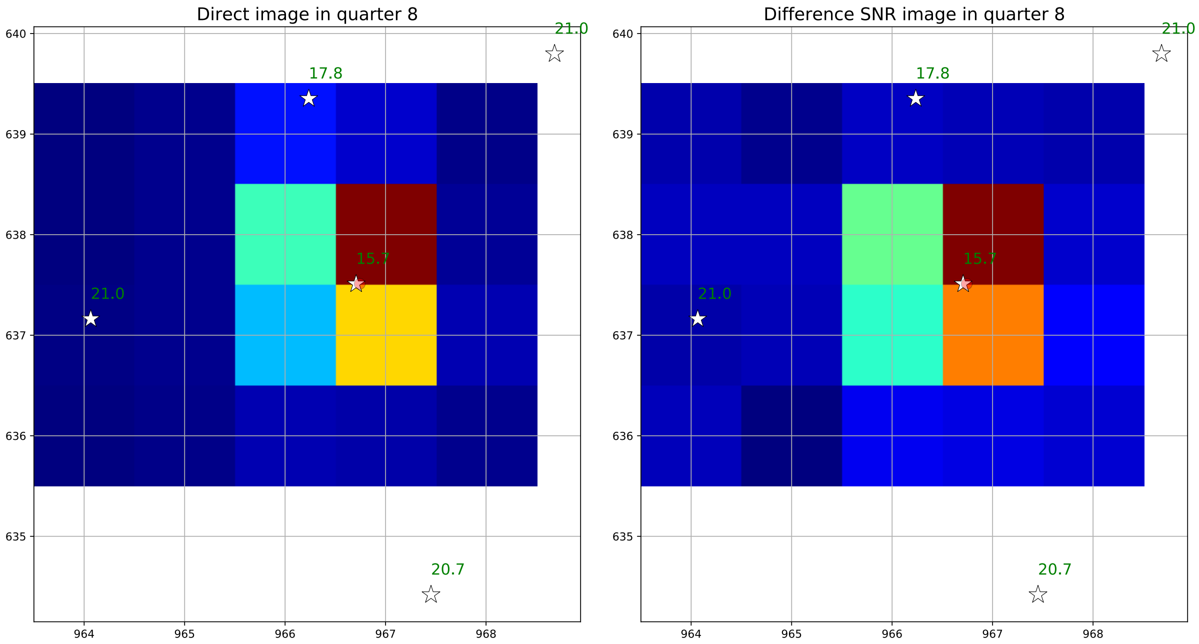

Supplementary Figure 5 | Observed *Kepler* image of KIC-7906827 during quarter 8. Left: Observed average out-of-transit image. Right: Observed difference image normalised by pixel-by-pixel uncertainty. The star symbols are the proper-motion-corrected *Gaia* star positions, and the semi-transparent red circle is the non-proper-motion-corrected *Gaia* position of the target star.

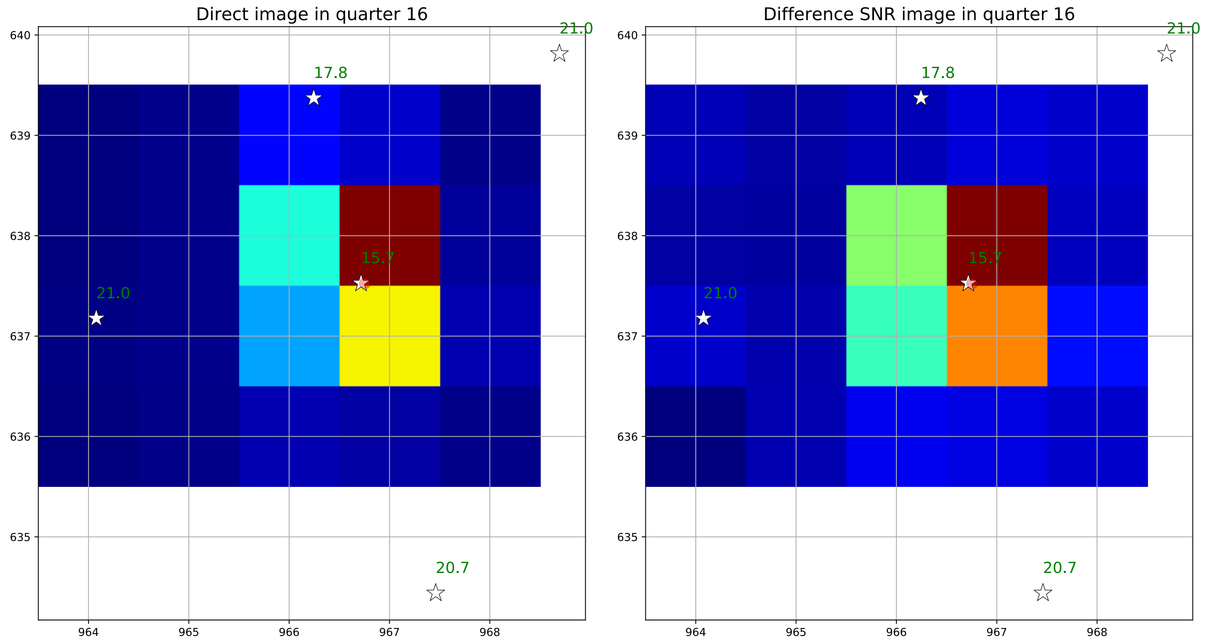

Supplementary Figure 6 | Observed *Kepler* image of KIC-7906827 during quarter 16. Left: Observed average out-of-transit image. Right: Observed difference image. The star symbols are the proper-motion-corrected *Gaia* star positions, and the red circle is the non-proper-motion-corrected *Gaia* position of the target star.

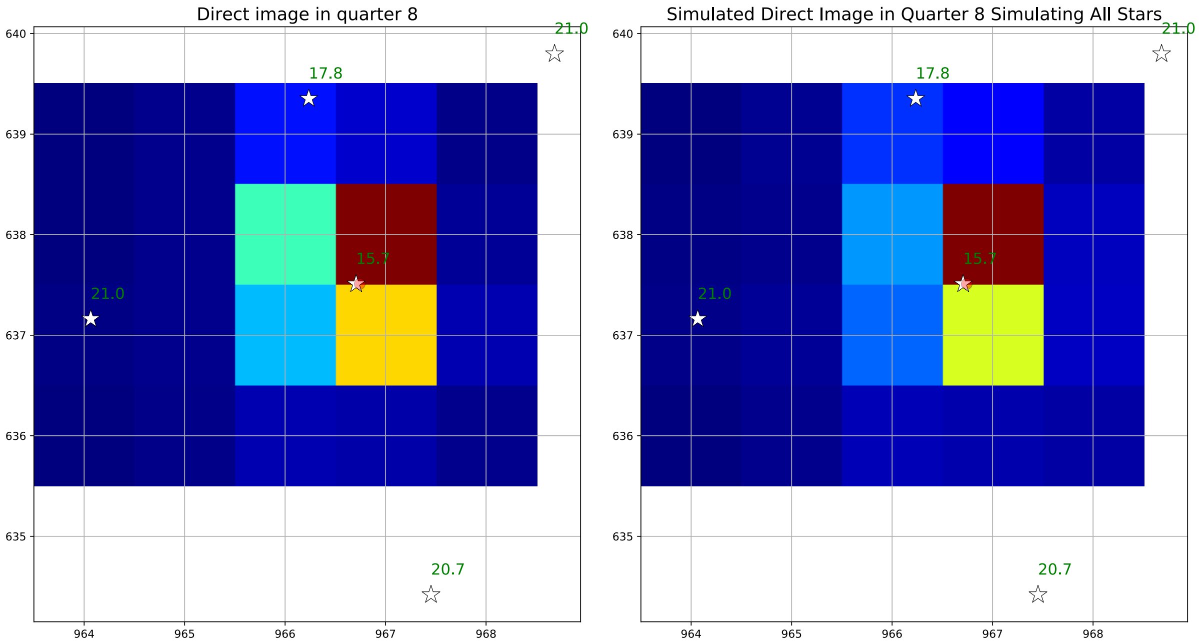

Supplementary Figure 7 | Comparison of the observed and simulated *Kepler* images of KIC-7906827 during quarter 8. Left: Observed average out-of-transit image. Right: Simulated average out-of-transit image.

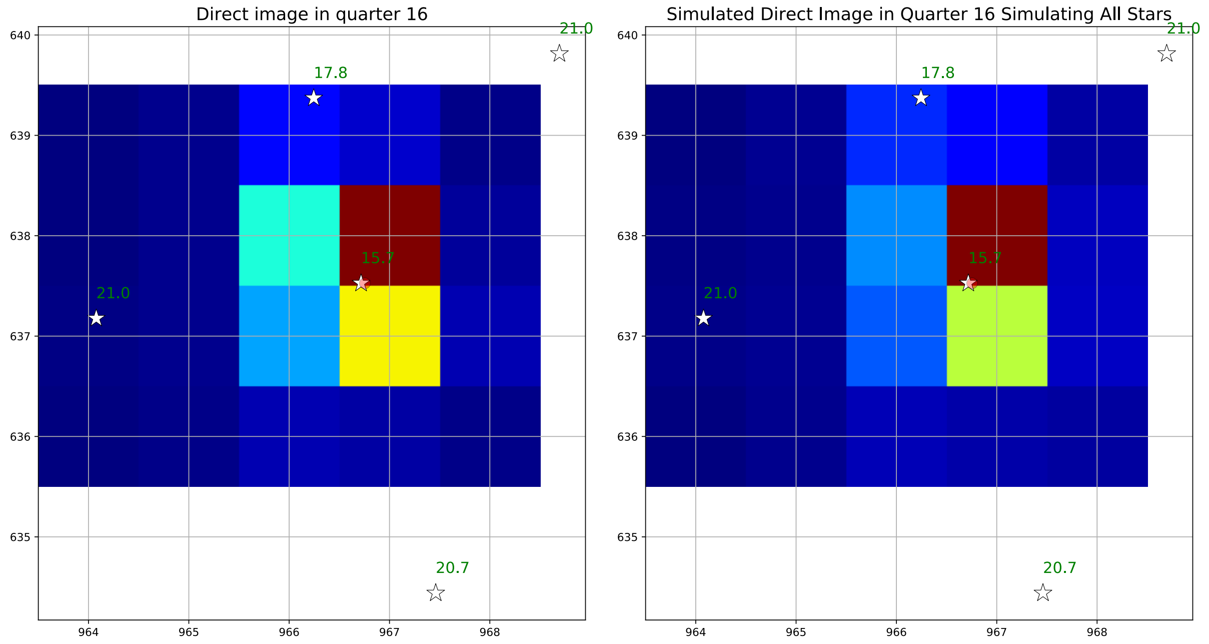

Supplementary Figure 8 | Comparison of the observed and simulated *Kepler* images of KIC-7906827 during quarter 16. Left: Observed average out-of-transit image. Right: Simulated average out-of-transit image.

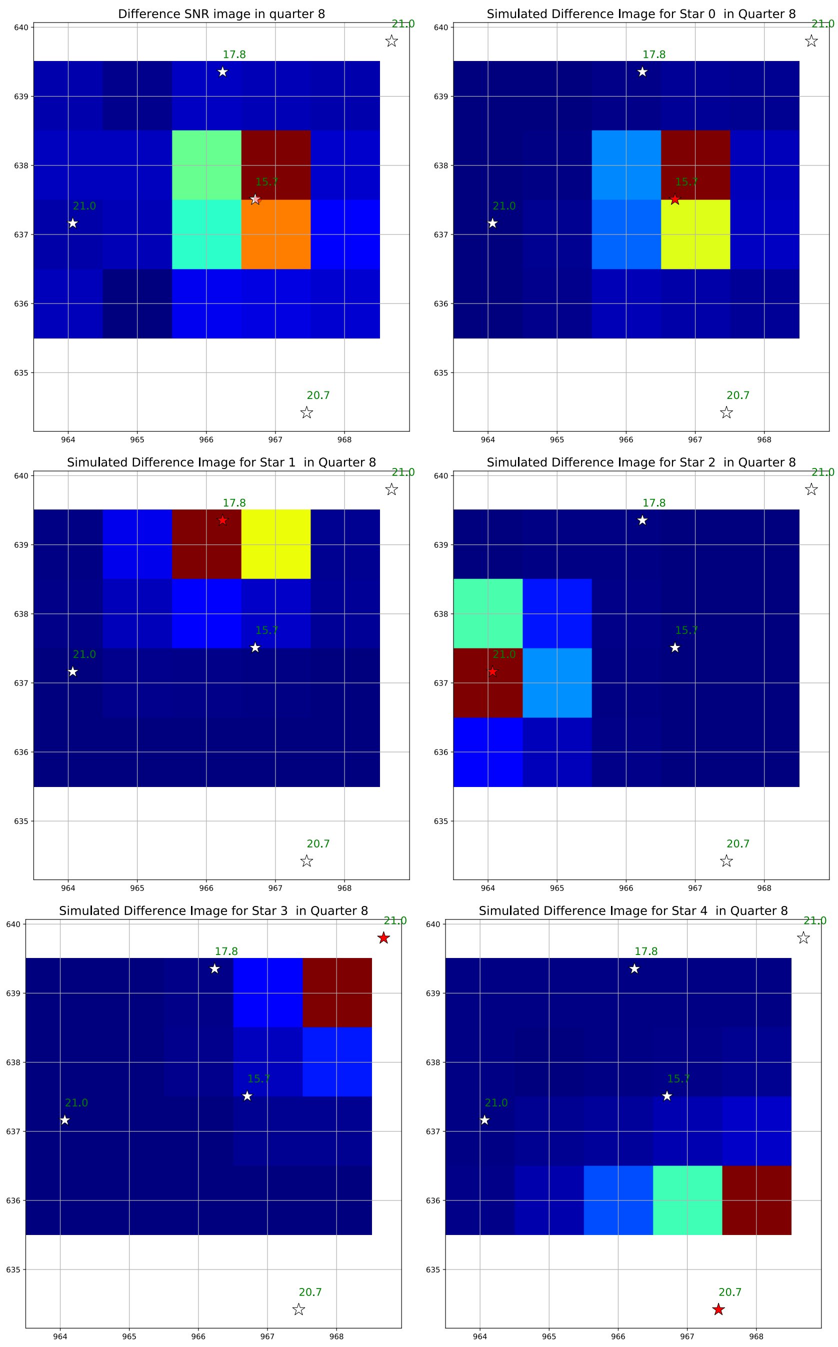

Supplementary Figure 9 | Simulated difference images of the KIC-7906827 postage stamp during quarter 8. Simulated difference images for the various stars compared with the observed difference image. The simulated star is shown with the red star symbol.

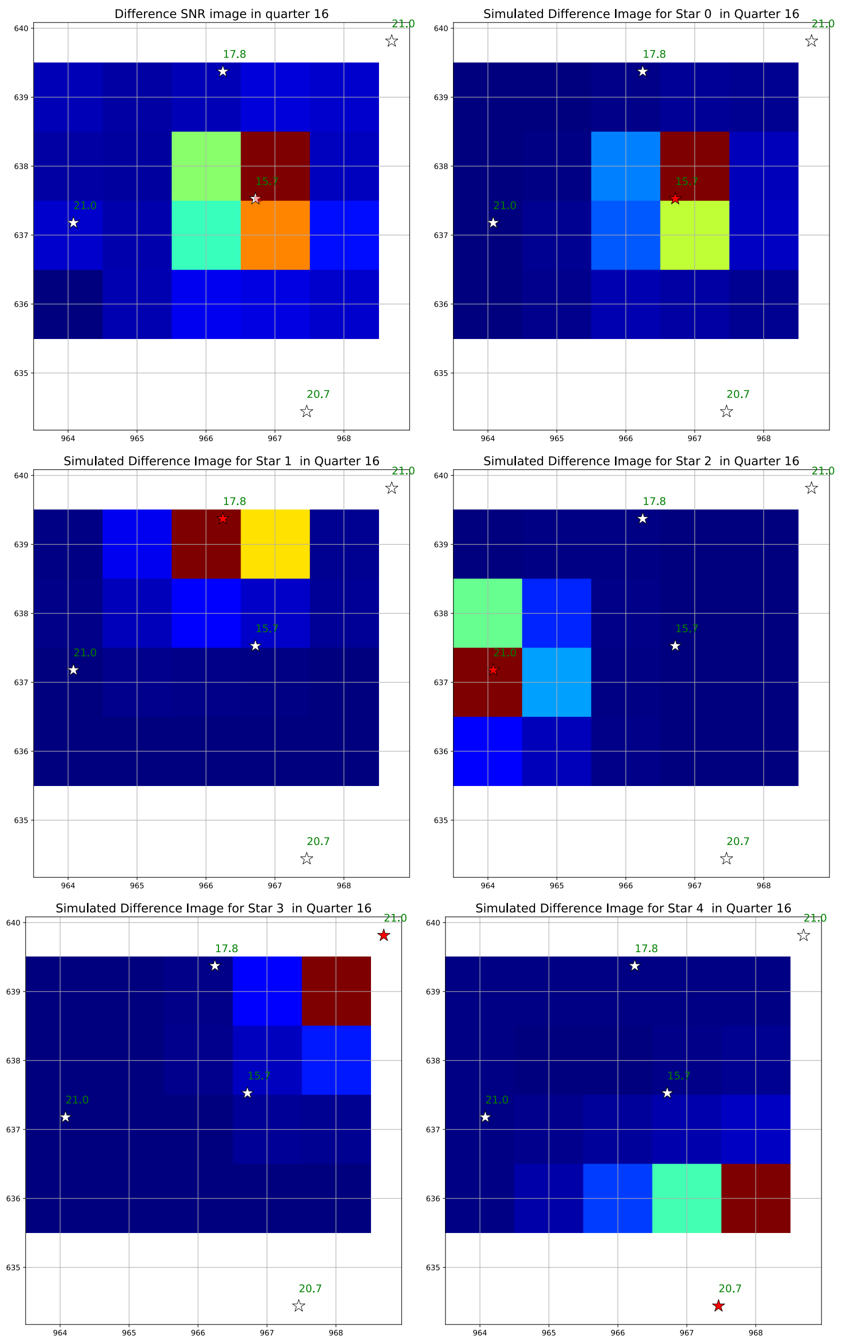

Supplementary Figure 10 | Simulated difference images of the KIC-7906827 postage stamp during quarter 16. Simulated difference images for the various stars compared with the observed difference image. The simulated star is shown with the red star symbol.

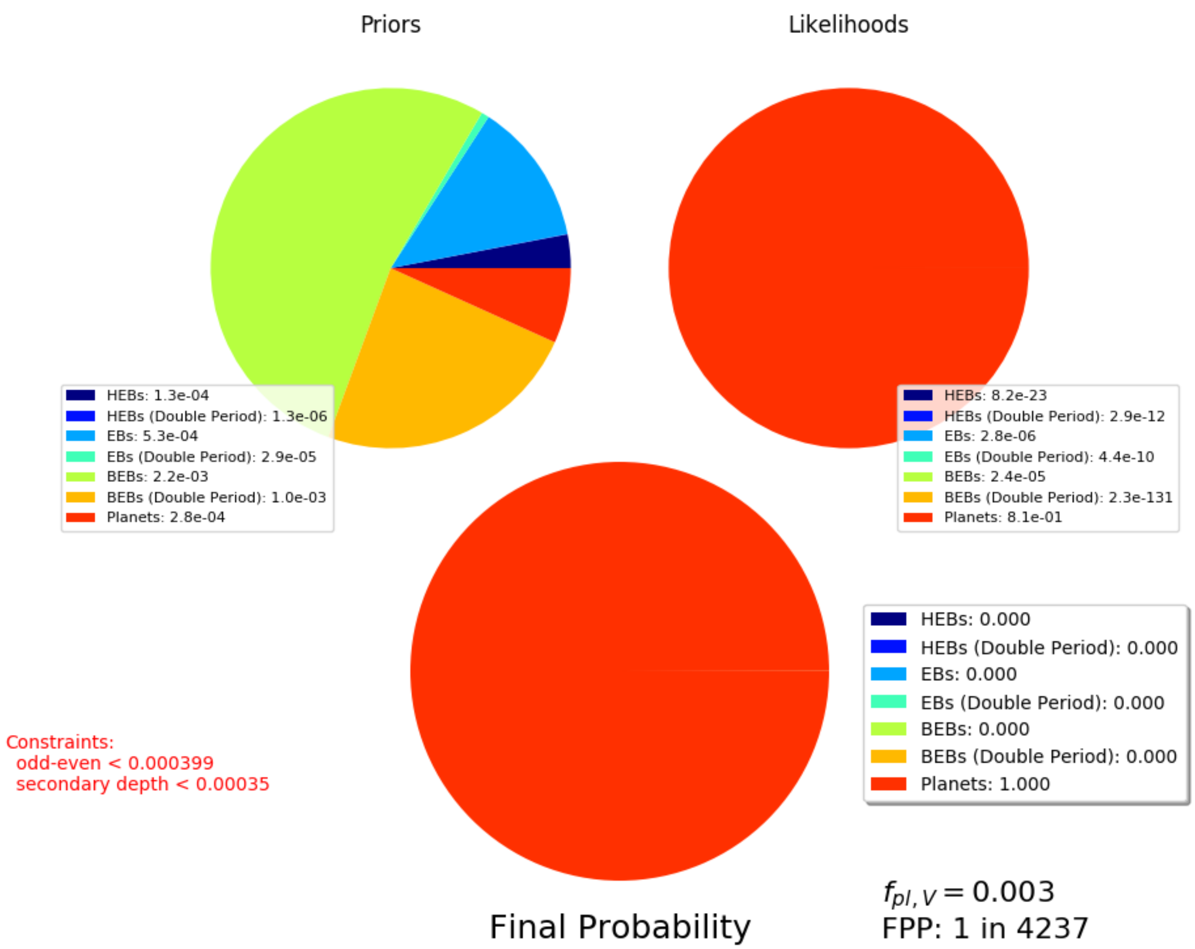

Supplementary Figure 11 | Auto-generated output summary figure from `vespa` **for KIC-7906827.01.** Pie-charts showing the relative odds of various astrophysical models for the observed transits of KIC-7906827. Top-left shows the adopted priors, based on the stellar properties and position. Top-left shows the likelihood governed by the transit light curve morphology. Bottom shows the posteriors odds, from which we conclude high confidence the planetary nature of KIC-7906827.01 (henceforth Kepler-1708 b).

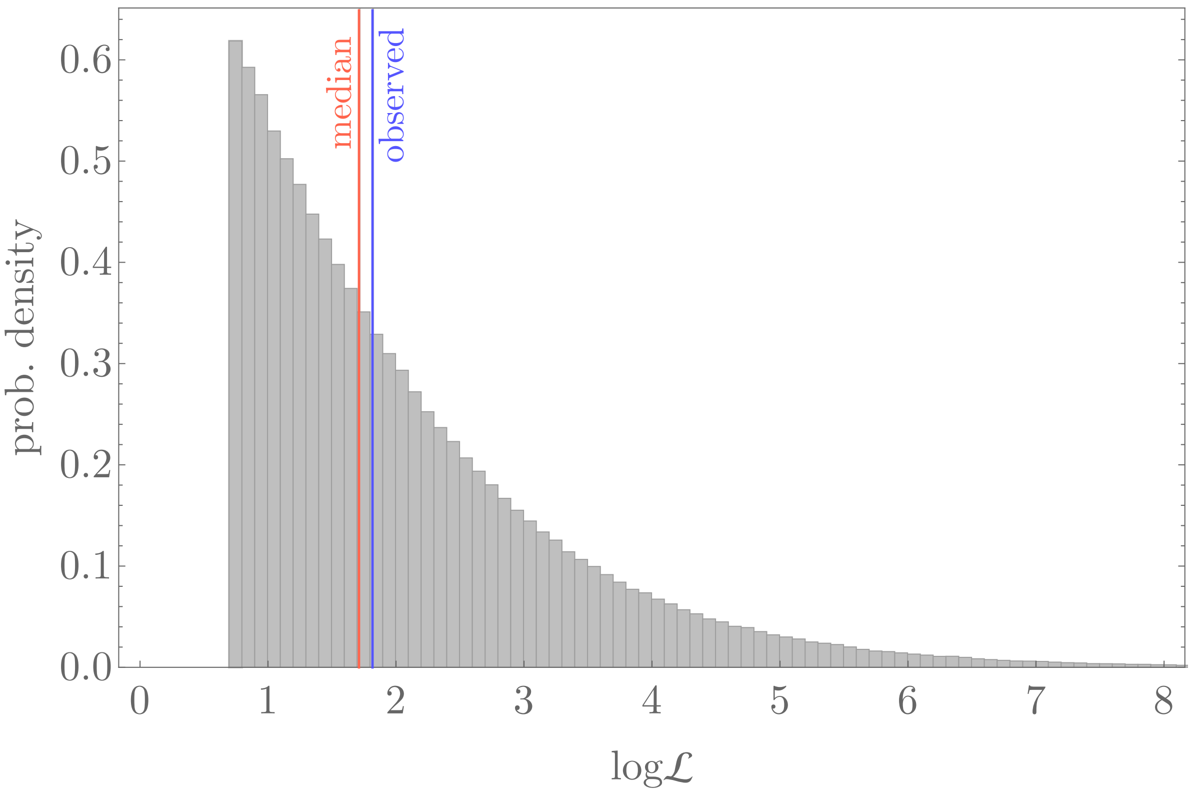

Supplementary Figure 12 | Log-likelihood distribution of two randomly sampled exomoon transit times. Through Monte Carlo simulation and geometric arguments[107], the distribution of exomoon transit times is expected to follow an arcsin distribution. Here, we evaluate the log-likelihood of observing the two transit times of Kepler-1708 b-i under this assumption (blue line), which is compared the distribution expected for random samplings. This reveals that the observed times are fully consistent with the expected behaviour.

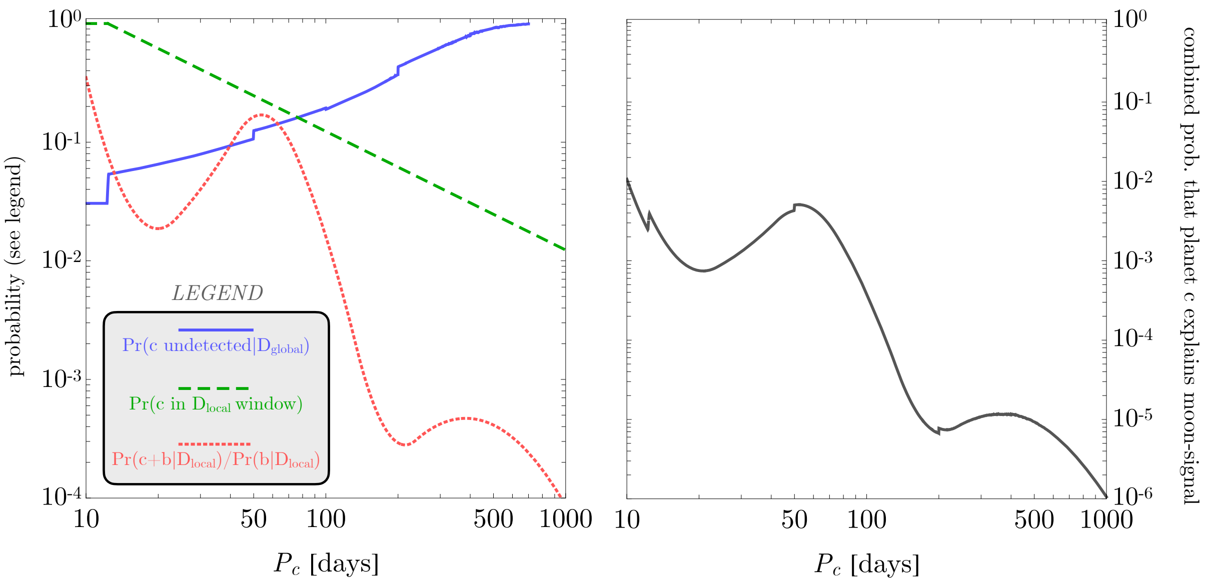

Supplementary Figure 13 | Probability of a second transiting planet explaining the moon-like deviations observed for Kepler-1708 b. Left: The probability of the three independent necessary criteria: 1) planet c evades detection by *Kepler* (blue solid). 2) planet c transited during the transit window of Kepler-1708 b (green dashed). 3) planet b+c model is statistically favoured over planet b alone (red dotted). Right: Combined probability using all three effects.

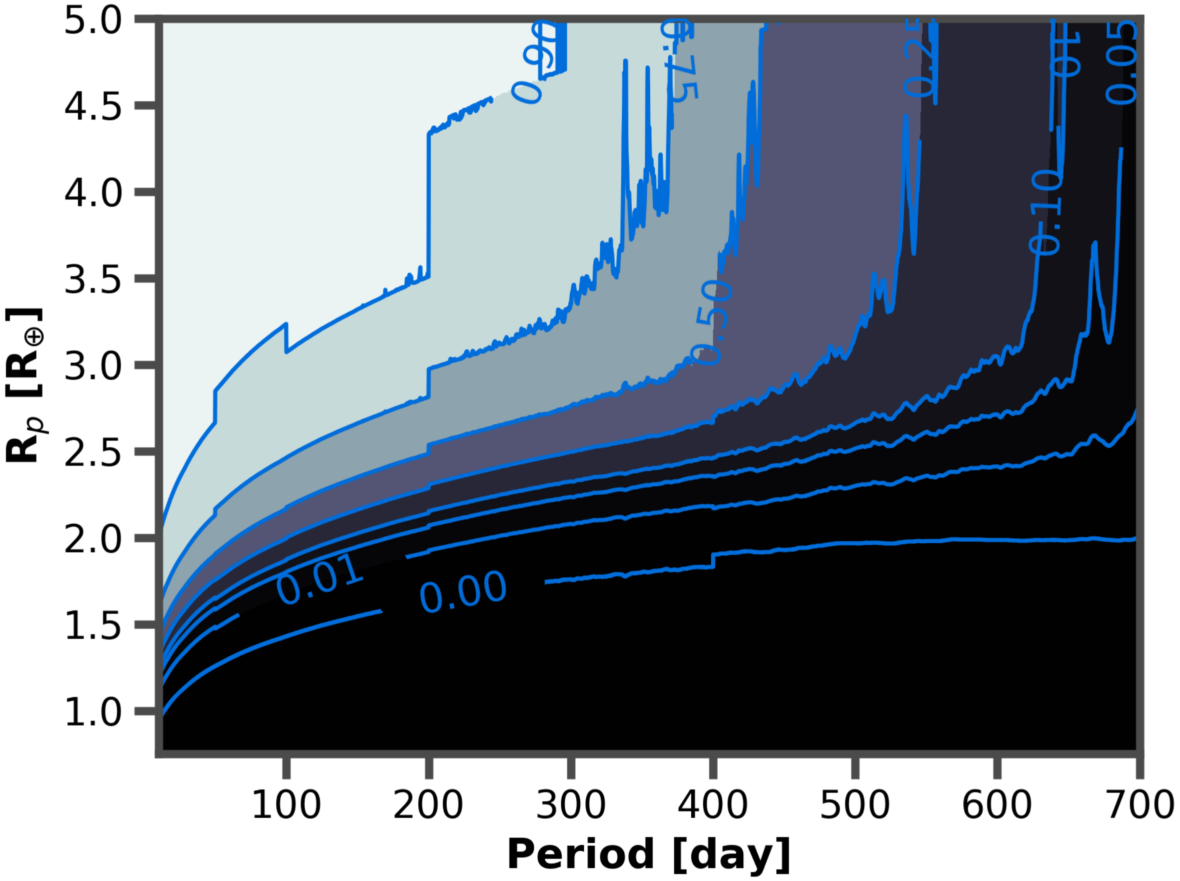

Supplementary Figure 14 | Detection probabilities for a second transiting planet orbiting Kepler-1708. Detection probability contour for a planet of a given orbital period and planetary radius (R_P) for the target Kepler-1708. Results are calculated for the DR25 *Kepler* planet candidate catalog and include the effects of the vetting procedure.

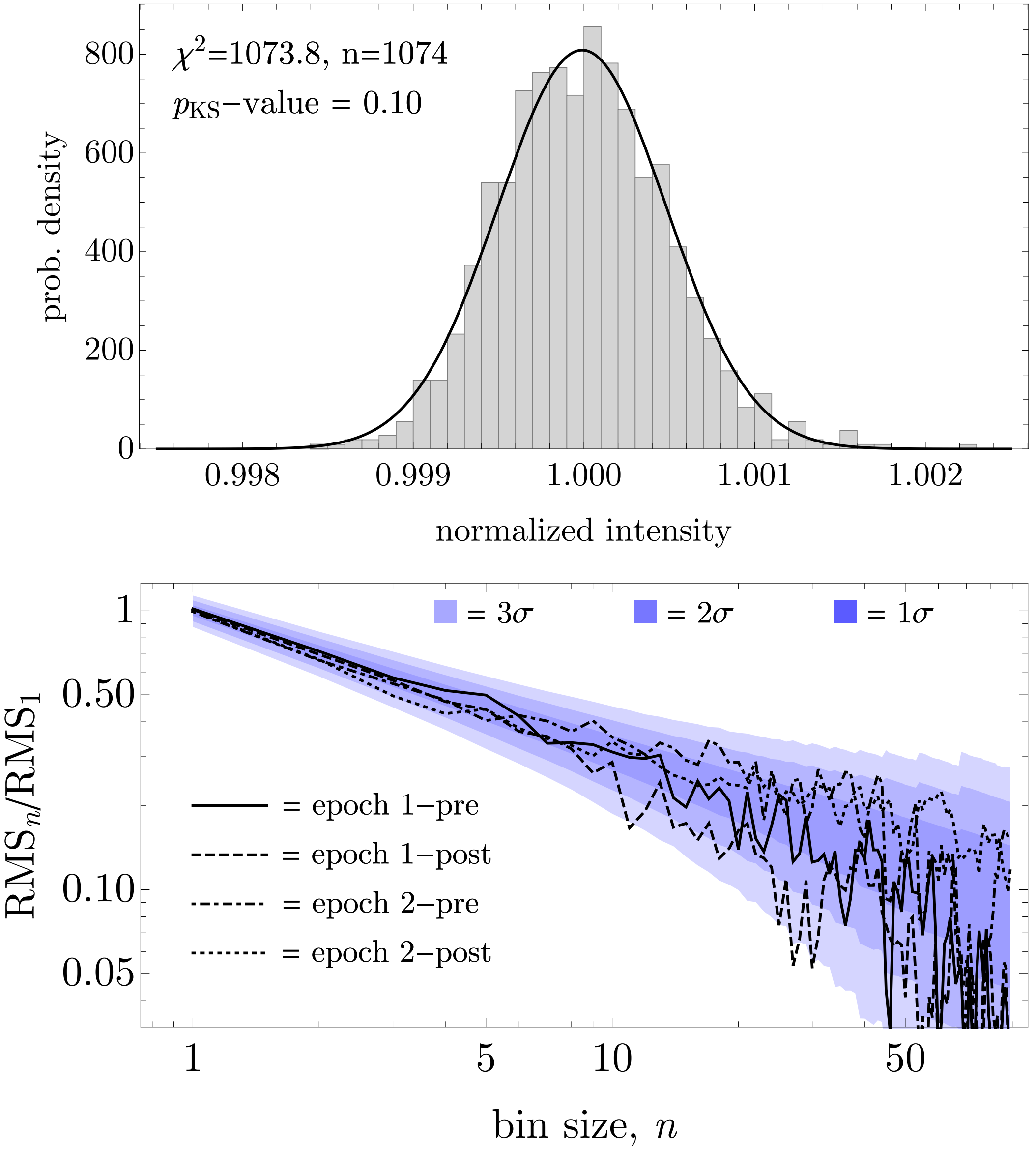

Supplementary Figure 15 | Tests for the Gaussianity on the local out-of-transit photometry of Kepler-1708 b. Top: Histogram of the method marginalised detrended photometry (excluding the transits) of Kepler-1708. The plotted function is not a fit but the expected form if the data were normally distributed and governed by the measurement uncertainties. Bottom: RMS vs bin test of the data, showing the four relevant regions with separate black lines and the expected range for Gaussian statistics in blue.

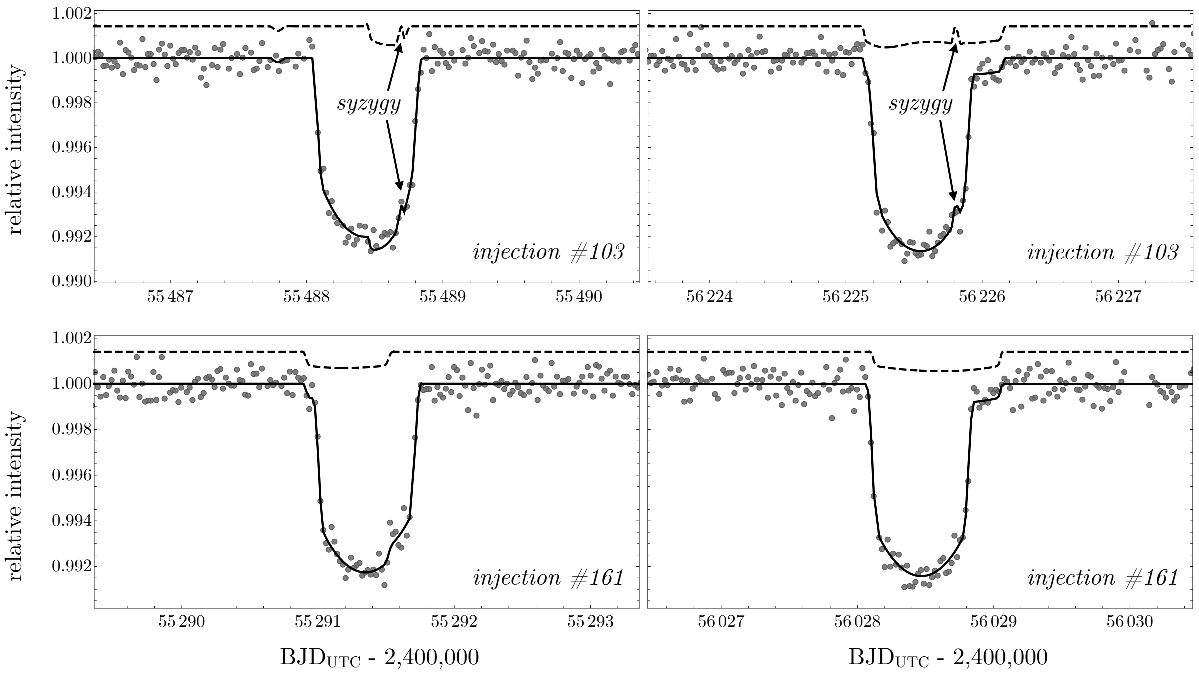

Supplementary Figure 16 | Transit light curves of the two false-positives found in our injection-recovery exercise for Kepler-1708 b-i, with the moon components shown as dashed lines above. Top shows injection #103 and bottom shows injections #161, both of which include two planetary transits (two columns). Both cases correspond to positive radii and have "strong evidence" via the Bayes factor tests. The spurious moon of injection #103 requires an inclined ($\sim 45°$) moon (causing the slope in its trough due to limb darkening effects) and a short-period of 36 hours (leading to syzygies highlighted).

[ht]

	Quarter 8	Quarter 16
Recovered transit depth	7.78×10^{-3}	7.68×10^{-3}
Distance from target star (pixels)	0.0146 ± 0.0154	0.0192 ± 0.0153
Distance from target star (arcsec)	0.058 ± 0.061	0.076 ± 0.061
3σ circle area (square arcsec)	0.107	0.104
Blend probability	2.62×10^{-6}	2.56×10^{-6}

Supplementary Table 3 | Summary of the results from our MCMC PRF blend analysis.
